# Novel phages of healthy skin metaviromes from South Africa

**DOI:** 10.1038/s41598-018-30705-1

**Published:** 2018-08-16

**Authors:** Leonardo Joaquim van Zyl, Yoonus Abrahams, Emily Amor Stander, Bronwyn Kirby-McCollough, Roland Jourdain, Cécile Clavaud, Lionel Breton, Marla Trindade

**Affiliations:** 1 Institute for Microbial Biotechnology and Metagenomics (IMBM), University of the Western Cape, Robert Sobukwe Road, Bellville, Cape Town, South Africa; 2 L’Oréal Research and Innovation, 1 Avenue Eugène Schueller, 93600 Aulnay sous Bois, France

## Abstract

Recent skin metagenomic studies have investigated the harbored viral diversity and its possible influence on healthy skin microbial populations, and tried to establish global patterns of skin-phage evolution. However, the detail associated with the phages that potentially play a role in skin health has not been investigated. While skin metagenome and -metavirome studies have indicated that the skin virome is highly site specific and shows marked interpersonal variation, they have not assessed the presence/absence of individual phages. Here, we took a semi-culture independent approach (metaviromic) to better understand the composition of phage communities on skin from South African study participants. Our data set adds over 130 new phage species of the skin to existing databases. We demonstrated that identical phages were present on different individuals and in different body sites, and we conducted a detailed analysis of the structural organization of these phages. We further found that a bacteriophage related to the *Staphylococcus capitis* phage Stb20 may be a common skin commensal virus potentially regulating its host and its activities on the skin.

## Introduction

Bacteriophages (viruses that specifically infect bacteria) are the most abundant biological entities on the Earth with an estimate of ten phage particles to every bacterium and a total of 10^31^ phage particles^1^. Phages have been shown to be key drivers of microbial population dynamics, as well as affecting food chains^2^. In nature, this can affect biogeochemical cycling of nutrients, drive host diversification and cause the ultimate collapse of trophic structures^2,3^. The advent of powerful tools such as high throughput next generation sequencing (NGS) now allows these phage communities to be studied as a whole (metagenomic), removing the traditional requirement to be able to culture the bacterial hosts in the laboratory. Culture-independent methods have become even more important as we have discovered that the vast majority of bacteria have not been, or currently cannot be, cultured under standard laboratory conditions^4^.

Since 2008, NGS has been directed at gaining a better understanding of the interactions between humans and their associated microbial communities^5^. One portion of the microbial community that has not received as much attention with respect to its influence on these communities are the bacteriophages^6^. Metavirome studies on the oral cavity, gut and vagina have been performed and links to disease states in these body sites have been found, however these studies have not been extended to the skin^7–17^. Some skin metagenomic studies have investigated the portion of their data that reflects the viral diversity in their samples^18,19^, however it is only recently that the presence of phages, and their possible influence on healthy skin microbial populations, has been specifically investigated^20^. The skin virome is also thought to become one of the new areas for exploration using metagenomic techniques as only these can capture the genetic variations whether in bacteria or viruses, that may play a role in infection, immune evasion or widening of host tropism^21^.

The few skin metavirome studies conducted have looked at the overall picture of which phages are present on the skin and tried to establish global patterns of skin-phage lifestyles. Skin metagenome and metavirome studies have indicated that the skin virome is highly site specific and shows marked intra- and interpersonal variation. They have not assessed the presence or absence of individual phages nor has the genomic detail associated with the identified phages been investigated which could potentially uncover the role they play in skin health. Therefore, a knowledge gap remains with respect to how these phages shape skin microbial communities and could be the cause of, or treatment for, certain skin diseases through their effects on the microbial population^22^. The link between microbial dysbiosis and disease states has been well established for the gut and it is expected that similar links will be found for the skin microbial population^23,24^. A specific example of why it would be important to better understand these interactions, is that it has been demonstrated that *Staphylococcus aureus* strains devoid of prophages ɸNM1-4 are either incapable of infection or have altered virulence patterns and that phages ɸ80α and ɸJB can mediate transfer of methicillin-resistance encoding plasmids between *S*. *aureus* strains^25,26^. Phage therapies have been investigated in mouse models for the treatment of *Staphylococcus* skin infections with some success^27^, and it is widely thought that this is a potential avenue for the treatment of a variety of bacterial infections.

The modular nature of bacteriophage genomes and the limited robustness of existing databases limit the understanding of phage genome architecture and can lead to annotation as “multiple hits” instead of a single species during taxonomic assignment during metagenomic data analysis^20^. Adding new phage genomic information, especially complete phage genomes, to these databases will help with future studies of these skin microbial populations. Most of the published studies have been performed on American, European and Asian skin types^19,20,28^, whereas our study represents the first that investigates phages on skin from African inhabitants (participants were predominantly of African and mixed ancestry).

Here, we took a semi culture-independent approach to better understand the potential composition of phage communities on skin from six South African male study participants, sampling the scalp, axilla and forearm, which are representative of the three main ecosystems of the skin (sebaceous, moist and dry respectively). We looked at the phage genome architectures of some of the phages that infect bacteria from the skin which may have clinical relevance and how they differ from those currently known.

## Materials and Methods

### Sample collection, processing and phage enrichment

Sampling was performed in accordance with the ethics policy of the University of the Western Cape. Samples were collected from 6 male subjects between 18 and 30 years of age and labelled accordingly (Supplementary Table S1). Volunteers gave their written informed consent prior to the start of study and the study was conducted in accordance with the University of the Western Cape ethics committee guidelines and their approval (Registration number 14/9/60). Volunteers were given a standard bland shampoo to be used three times a week at home. The last shampoo was performed three days before sampling. Likewise, they received a shower gel for their body. The last shower was performed 24-h prior to sampling. Volunteers were requested not to use any other products to wash their hair, scalp and body during the 2-week wash-out period or using deodorant/antiperspirant within 7-days prior to sampling. Three skin areas were sampled: outer forearm (F; dry), scalp (S; sebaceous) and axilla (AX; moist). For all three sites, samples were collected using Floq’ed swabs (nylon handle; FLOQSwabs Copan, Murrieta, USA). The sampling buffer consisted of 150 mM sodium chloride (Sigma-Aldrich S7653, Darmstadt, Germany) and 0.1% (vol/vol) Tween 20 (Sigma-Aldrich P7949). The solution was made using sterile DNA/RNA free water (Gibco, Waltham, USA). Aliquots (5 ml) of buffer were aseptically transferred into 15 ml Falcon tubes. For scalp sampling, a sterile swab was soaked in the collection buffer and rubbed on the scalp surface along a line made by parting the hair and making four passages. The swab was immediately immersed into the saline buffer and shaken vigorously. These steps were performed 8 times, parting the hair each time to cover a different track, to cover a surface of 16 cm^2^ and the swab returned to the buffer for storage. For forearm and axilla sampling, a sterile swab was soaked in the collection buffer and rubbed on the body surface for 60 seconds to cover a surface of 16 cm^2^. The swab was immediately immersed in the saline buffer and shaken vigorously. Then the 60 seconds rubbing step was repeated once and the swab returned to the buffer for storage. For phage enrichment, 250 µl of the undiluted original sample was used to inoculate 10 ml of MRS (ATCC Medium: 416 Lactobacilli MRS Agar/Broth, Sigma-Aldrich S69966, Darmstadt, Germany) broth in a 15 ml Falcon tube. These were incubated upright with loosened caps at 30 °C for 16 hours shaking at 250 RPM, prior to adjusting the temperature to 37 °C for another 16 hours.

### Phage DNA isolation and sequencing

For phage DNA preparation, 6 ml of supernatant from the enrichment broth was first passed through a 0.45 µm syringe filter (GVS FLAS25AC4550) to remove eukaryotic cells and bacteria. 50 µl sterile 100 mM Tris-HCL pH 8 (10 mM final concentration) and 5 µl RNAseA (Fermentas, Vilnius, Lithuania, #EN0531, 10 mg/ml) was added to the filtrate. After incubating at room temperature for 10 min, 600 µl of 10x DNaseI buffer was added (100 mM Tris-HCl pH7.5, 25 mM MgCl_2_, 1 mM CaCl_2_). Finally, 10 µl of DNaseI was added (Fermentas; #EN0521, 1U/µl) and the mixture incubated at 37 °C for 16 hours. A 10 µl aliquot of the #10 sample for each skin area was removed to which *Geobacillus thermoglucosidasius* genomic DNA was added to determine whether the DNaseI was active under the sample conditions and the mixture incubated at 37 °C O/N. The result was visualized on an agarose gel. To further confirm the absence of contaminating bacterial DNA, all samples were screened by PCR with primers that target the bacterial 16S rRNA gene using primers E9F: 5′-GAGTTTGATCCTGGCTCAG-3′^29^ and U1510R: 5′-GGTTACCTTGTTACGACTT-3′^30^. 25 µl PCR reactions were set up using Phusion DNA polymerase (Thermo Scientific, Waltham, USA) and the high G + C buffer supplied. The manufacturer’s protocol was followed and 10 µl of sample was used as template. An annealing temperature of 52 °C was used. A spiked control was included to determine whether the constituents of the sample would inhibit PCR. Phage DNA was extracted using standard phenol/chloroform extraction as described previously^31^. The DNA was re-suspended in 20 µl TE buffer pH 8.0 and stored at room temperature. Sequence library preparation of the phage DNA was performed with the Nextera XT kit (Illumina, Hayward, USA) with a 10% phiX v3 spike as per the manufacturer’s instructions (Preparation Guide, Part #15031942 Rev A May 2012) and the MiSeq Reagent kit V3 (600 cycle). One ng of uncloned, unamplified viral DNA was used to prepare one NexteraXT library per sample. The resultant libraries were sequenced using the Illumina MiSeq at the University of the Western Cape generating 2 × 300 bp reads per sample. The raw reads were trimmed (bases with a Q-score less than 36 were trimmed from the 3′ end) and demultiplexed at the sequencing facility.

### Assembly of read data, phage annotation and comparison

Read data was assembled using CLC Genomics Workbench version v7.5.1 (Qiagen, http://www.clcbio.com) with the length fraction set at 0.8 and similarity fraction at 0.9. Mismatch insertion and deletion costs were left at their default values. The “global alignment” and “update contigs” settings were enabled, while scaffolding was turned off. Word- and bubble size was set to automatic mode as well as paired distance detection. As we were expecting to recover complete phage genomes due to viral enrichment, only contigs above 2.5 kb were considered for analysis. The assembled contigs used in the analysis presented here were submitted to the GenBank database under accession numbers MF417837-MF417995. Reads from various metagenome and metavirome studies were mapped to our phage contigs using the built in “map reads to reference” function in CLC Genomics Workbench. The same length and similarity fraction parameters mentioned above were used for read mapping of these datasets to our phage contigs. Non-specific match handling was set to “ignore” and no mask was applied. Mismatch, insertion and deletion costs were left at the default settings. The SRR file names from metavirome and metagenome datasets used for read mapping against the contigs identified here are provided in Table S3 while the “Earth’s virome” contigs are available at http://portal.nersc.gov/dna/microbial/prokpubs/EarthVirome_DP/. All contigs recovered after *de novo* assembly were screened using Virsorter to identify true phage contigs among those generated after assembly using the Viromedb as selection criteria^32^. In parallel, contigs were uploaded to MetaVir 2 for analysis^33^. For more complete annotation of individual contigs PHAST (database update December 23^rd^, 2016), which includes prophage sequences, and MetaVir2 were employed together with manual curation in CLC Genomics Workbench^34,35^. For those contigs not recognized as phage or prophage by VirSorter, manual inspection of contigs searching for the presence of “viral keywords” (terminase, capsid-, head-; tail-, portal-, sheath-, tape measure-, phage replication-protein) was performed to ensure that no genuine phage contigs were missed. For those contigs where no “viral keyword” genes could be identified, the relation to phage was estimated based on the relation of encoded proteins to known phages using the MetaVir 2 parameters (comparison against RefseqVirus with a threshold of 10^−3^ on e-value and 50 on bitscore). All contigs identified as phage or prophage by VirSorter were used in downstream analysis. For taxonomic classification of the phage contigs, MetaVir 2′s annotation was used and Kronatools was used for visualization of taxonomic composition^36^. The taxonomic affiliation was used to generate a network for visualization of the presence or absence of related phages in the various samples performed with Cytoscape v3.4.0 (http://www.cytoscape.org/). Phage genomes were compared using Easyfig^36^. Using the protein sequences identified by MetVir 2 and PHAST as well as the nucleotide sequence of the contig GenBank formatted files are created using Sequin (https://tinyurl.com/y7g3rd9c) then manually edited in DNA Master (http://cobamide2.bio.pitt.edu/computer.htm) and then used for comparison in Easyfig. Manual pairwise alignments were performed, and the final figures constructed to display the closest relationship between pairs of phage genomes. Cluster analysis was performed with Primer v6, following conversion of phage taxonomic affiliation to presence/absence data^37^. Amino acid sequence concatenation was performed using SequenceMatrix v1.8^38^. Sequence alignment for phylogenetic tree construction was performed with the MUSCLE program included with the MEGA 6 software package^39^, and the tree constructed using the neighbour-joining method. Potential CRISPR sequences were identified through BLASTn against the CRISPR database on CRISPR finder using the default BLAST settings (E-value of 0.1 and a matching length of at least 70% the queried spacer size) and accessed on May 25th, 2017^40^.

### Phage microscopy

Prior to extraction of phage DNA, all samples were preliminary screened for the presence of phage particles by fluorescent microscopy. 10 µl aliquots of supernatant were transferred to sterile 1.5 ml Eppendorf tubes and 0.3 µl of undiluted SYBR GOLD was added and incubated at 80 °C for 10 min. 5 µl of the total volume was placed on a glass slide and examined under 1000x magnification using a Zeiss Axioplan II fluorescence microscope fitted with filter set 25. To further visualize phage particles, 1 ml of the initial enrichment culture was centrifuged at 5000 RPM for 10 min to pellet bacteria. The supernatant was centrifuged at 25000 × g for 2 hours in a fixed angle rotor to pellet phage particles. The pellet was re-suspended in 50 µl 100 mM ammonium acetate (pH 7.2) and left to re-suspend with shaking at room temperature for 16 hours^41^. The wash was repeated a second time with fresh buffer. The final pellet was re-suspended in 5 µl 100 mM ammonium acetate and visualized by TEM. Three microliters of each sample were pipetted onto carbon-coated 200-mesh copper grids and stained with 2% aqueous uranyl acetate. The samples were viewed using a LEO 912 Omega TEM at 120 kV (Zeiss, Oberkochen, Germany) housed at the University of Cape Town Physics Department. Images were collected using a ProScan CCD camera.

## Results and Discussion

### Overall description of the enriched skin metavirome and diversity analysis

**Figure 1:**
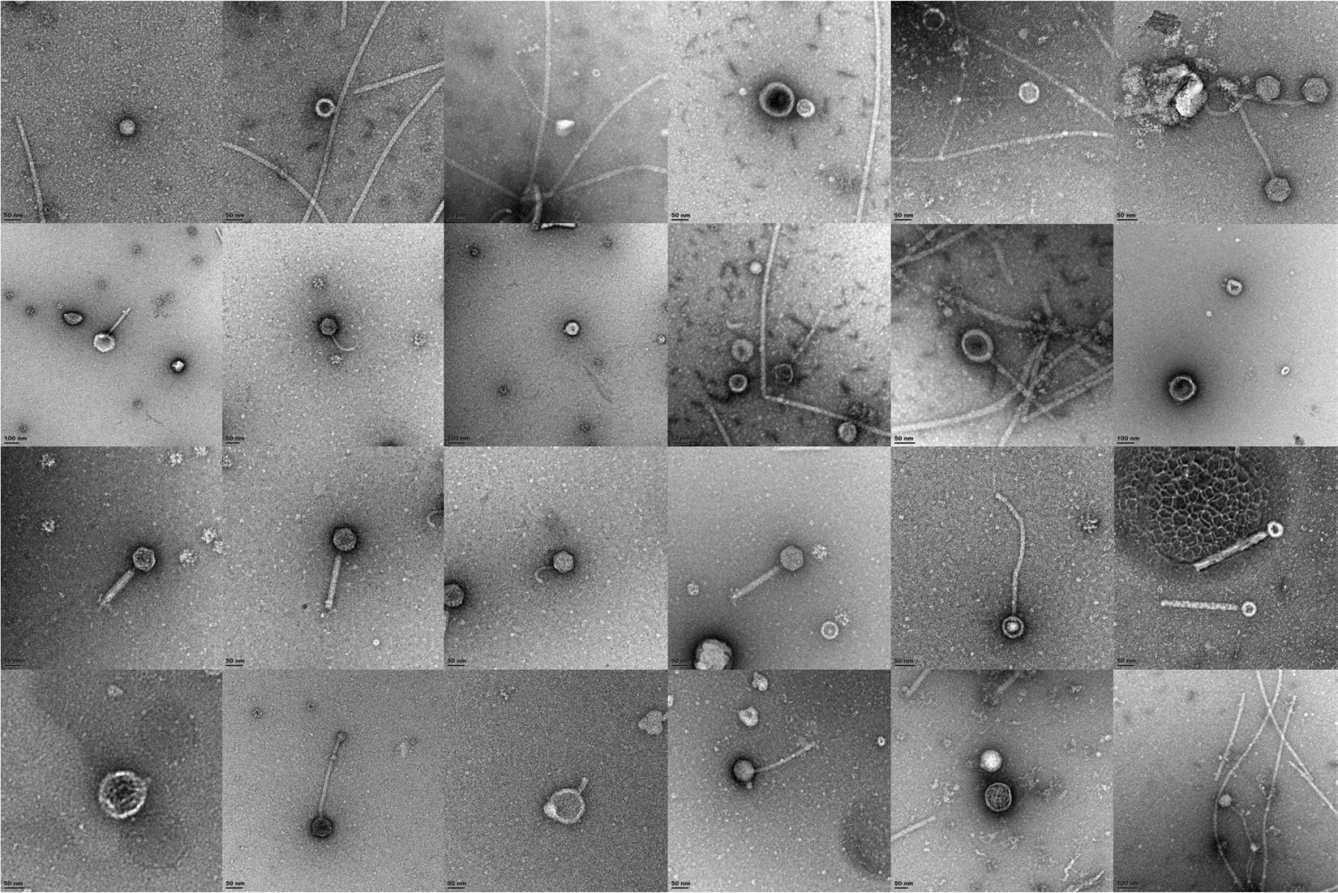
Phage particle morphology observed by TEM in enrichment culture supernatants.

The first step in the analysis of this enriched metavirome was the direct observation of phage particles from the enriched culture broth (Fig. 1). As expected, the samples were dominated by tailed phages with Sipho- and Myoviruses predominating based on tail morphology.

Of the 18 sequenced samples, two datasets were not informative (2S, 3AX, Supplementary Table S1). The total dataset contained 17.4 million reads, with 964554 reads per sample on average. Of the total read dataset, 8.3 million reads are represented by the contigs described here. Sample 2 S sequenced poorly, while 3AX consisted mainly of *Bacillus pumilus* genomic DNA. A total of 1391 contigs were analyzed with VirSorter, resulting in 115 (112 categorized as phages and 3 as prophages) contigs being retained as phages or prophages. Through manual inspection of the MetaVir 2 annotation of the contigs a further 44 contigs could be identified which were clearly phage related, resulting in 159 contigs ranging in size from 2852 bp to 141kbp (Supplementary Table S2) with four of these being detected as circular. Sequences related to *Siphoviridae* made up the bulk (73%) contrary to what was observed microscopically, *Podoviridae* made up 12% and *Myoviridae* 8% (Supplementary Fig. S1). The dominant host genera, based on phage taxonomy, was *Pseudomonas* (36%) followed by *Staphylococcus* (27%). Although these are common skin commensal bacteria, the bias probably also reflects their ability to grow in the media used. Although enriched, our metavirome data agrees with the Hannigan study^21^, showing that many of the *Staphylococcus* phages are related to *Phietalikevirus* including perhaps the orphan phage Stb20 (see below) and phiRS7 (Supplementary Fig. S1).

**Figure 2:**
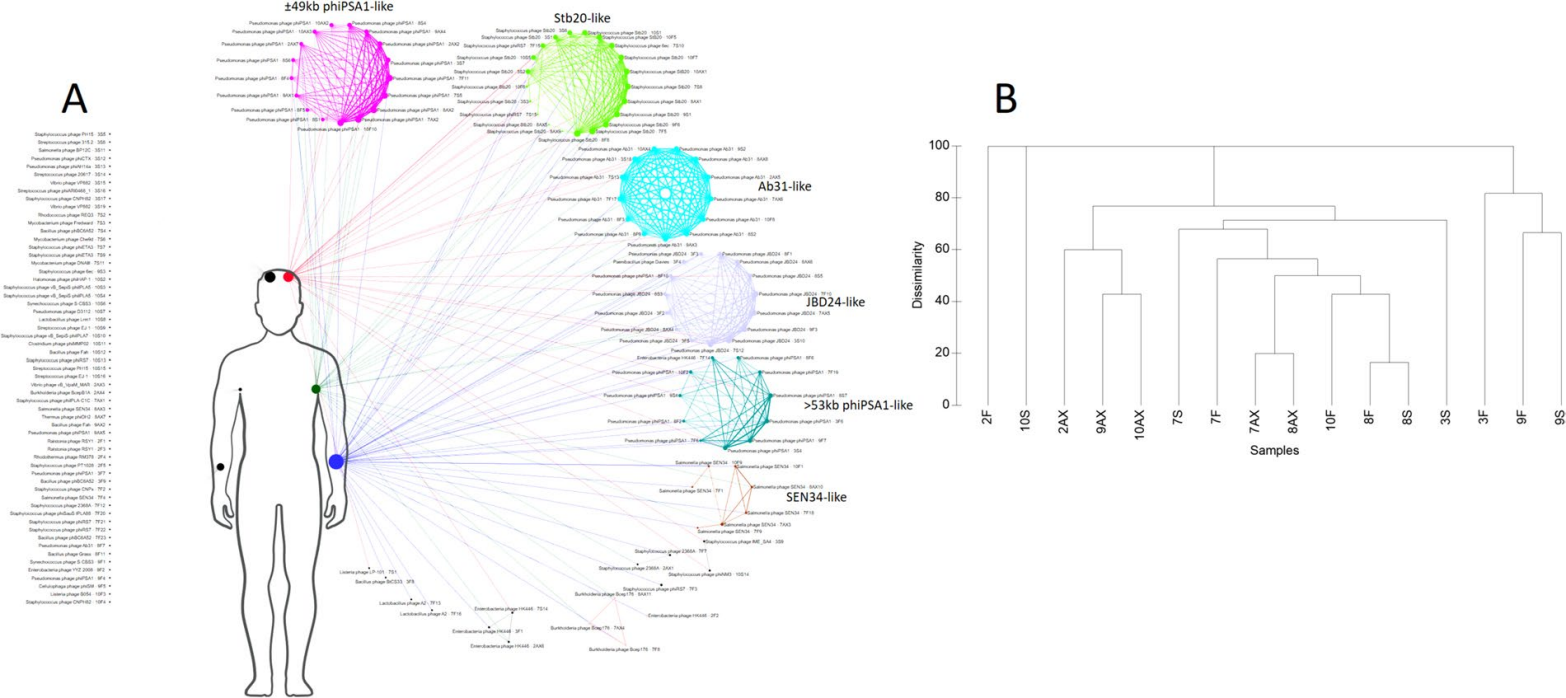
(**A**) Network analysis showing the distribution of unique (left side of human figure) and shared phages (right side) between the three body sites investigated. Node sizes scale according to the number of direct edges. Edges between nodes indicate a statistically significant relationship between the protein profiles of their viral genomes, with edge weight (darker = more significant) corresponding to their weighted similarity scores. (**B**) Cluster analysis of Bray-Curtis dissimilarity using presence-absence data for phages in the various samples.

To establish whether or not contigs were shared or unique to each sample, the contigs from all samples were analyzed using vConTACT (BLASTp cutoff e-value 10–80). This showed that many of these contigs share some similarity at the protein level even if not identical (Fig. 2A). The number of similar phages shared either between two or more individuals, or two or more body sites (99), was greater than the number of singletons (60). To determine whether those contigs identified as singletons, but with identical taxonomic affiliations, we looked if within one sample those contigs belong to single or multiple phage genomes (*Streptococcus* phage EJ-1 in 10 S or *Staphylococcus* phage phiRS7 in 7F), and we compared them with their closest relatives, using tBLASTx. Four sets of contigs (in 7S, 10S, and 3S), that may form part of the same genome, could be identified based on their mapping to a related genome and contig coverage (Supplementary Table S2). If these pairs are considered to belong to the same genome, this reduces the number of singletons to 56. There is the possibility that due to low similarity to related phages and the inability to join the contigs, more of these associations may have been missed here, making our number of unique phages an upper limit for this dataset. If contigs that cluster together according to the vConTACT analysis are considered to belong to a single phage “species”, the diversity of phages shared among individuals and body sites^11^ was much lower than the number of unique contigs/phages^42^ (Fig. 2A). This is in agreement with other studies that show high interpersonal and inter site variation in the viral metagenome. However, unlike other studies, our data points out that there is a small group of highly similar, if not identical, phages shared between most individuals and the three body sites analyzed. Cluster analysis based on taxonomic affiliation showed no clear correlation between either phages and individuals or phages and body sites. For some sites such as 8S and 10AX all contigs are shared with other individuals or body sites (Fig. 2B). Of the shared phages, most are *Pseudomonas* phage-like while of the *Staphylococcus* phages the Stb20-like phages were the most shared (Fig. 2A). Common contigs identified in different body sites from different individuals included phiPSA1-like phages, Stb20-like phages and vB_PaeP_Tr60_Ab31-like phages which will be discussed in detail below (Supplementary Table S2).

### Staphylococcus phages

**Figure 3:**
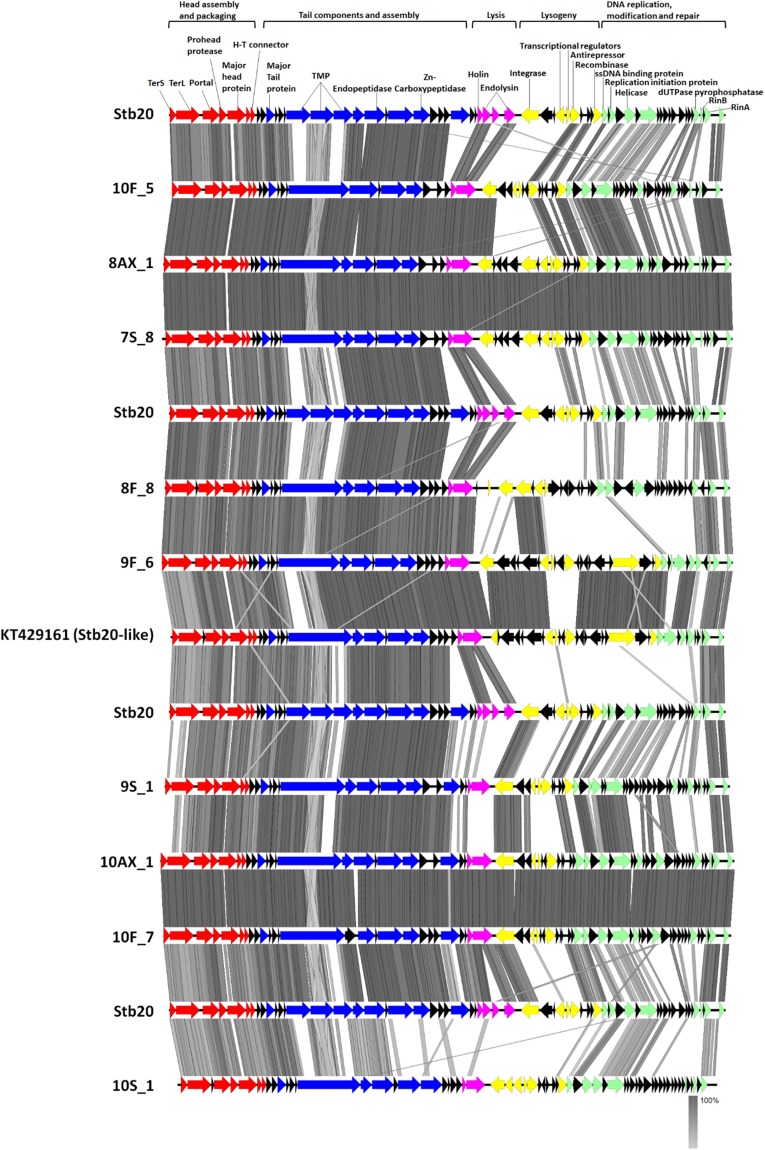
Comparison of the genomic structure of Stb20-like phage contigs >30 kb identified to the Stb20 phage genome.

When considering contig best BLAST hit classification (many or most proteins hit a particular phage genome), 12 contigs (2936bp-42205bp) showed top BLAST hit to *Staphylococcus capitis* infecting phage Stb20^43^. Related contigs, identified through vConTACT analysis, were also included in the Stb20-like group bringing the total number of Stb20-like contigs to 18 out of 159 (Supplementary Table S2 and Fig. 2A). The presence of Stb20-like phage in so many of the samples together with high coverage on some of these may indicate an easily inducible phenotype and suggests, that like some of the other shared phages, these are phages commonly associated with skin-related *Staphylococcus* species. In sample 10F, two Stb20-like phages could be identified. The reads were assembled with both length and similarity fraction at 95%. The two phage genomes assembled as four contigs with two of the contigs covered 2660-fold and 3321-fold (pair 1) while the other two were covered 920-fold and 833-fold (pair 2). For each contig pair, a “front half” and “back half” of an Stb20-like genome could be identified, leading to the conclusion that these were two Stb20-like phages which could not be assembled as only two contigs, likely due to similar sequences being present in the two genomes (sequences corresponding to ssDNA binding protein, replication initiator and helicase genes). Read mapping of 10F reads to the highly similar 10AX genome confirms the presence of reads covering these three genes in the 10F dataset and shows that they are identical to the two phages identified in this forearm sample. The front and back halves of each contig pair were combined to form pseudo-genomes, designated 10F_5 and 10F_7 with which to compare to the other identified genomes (Fig. 3). This finding would suggest that either there was one host present carrying two variants of an Stb20-like phage or two different hosts, each with its own variant. This also serves to show that on one individual, more than one species of the phage can be found, and more specifically, in one location on the body.

Phylogenetic analyses using the concatenated, terminase small and large subunit, portal protein, major capsid protein, major tail protein, tape measure protein, tail fiber protein, and tail endopeptidase sequences showed that there were two distinct clades, (Supplementary Fig. S2). Alignment of the 3 Stb20-like contigs identified from participant 7 demonstrated that each was unique. The 10AX_1 contig is identical to 10S_5 and is highly similar to 10F_7, showing that the same phage occurs on all three body sites sampled for the same individual. There are also three instances of different versions of the phage being present on one individual (8F_8/8AX_1; 9F_6/9S_1; 10S_1/10F_5/10AX_1/10S_5). One of the genomes identified here (9F_6), appears to be most closely related to the “Stb20-like” phage (KT429161) and shows the isolation of this phage geographically distant from its original place of identification (Russia); a good indication of the wider distribution of this phage. Unique to 9F_6 and the “Stb20-like” phage is an ATPase/partitioning-like protein that may play a role in stable inheritance of the phage or may play a regulatory role^44^.

Since the “back halves” of the Stb20-like genomes show substantial variation, it was of interest to determine which phages they are most similar to. Using the results from the MetaVir 2 annotation of these contigs, it appears that 7S_8, 8AX_1, 8F_8, 10AX_1, 10F_5 and 10F_7 have a back half with high similarity to a mixture of the two *S*. *epidermidis* infecting phages CNPH82 and vB_SepiS_phiIPLA5/7 with top BLASTp hits with total BLAST scores ranging from 52 to 815. The back half of 9S_1 appears mostly related to CNPH82 (total BLAST scores 97–221), while that of 9F_6 and the “Stb20-like” phage appears mostly vB_SepiS_phiIPLA5/Stb27-like (total BLAST scores 100–1222). Contig 10S_1 is the exception with a “back half” mostly related to the *Staphylococcus saprophyticus* phiRS7 phage (total BLAST scores 67-800). This would suggest that the Stb20-like phages identified here represent hybrids of Stb20 and *Phietalikevirus*-es (CNPH82 and vB_SepiS_phiIPLA5). It could be argued that these are sequencing and contig assembly artefacts. Even though phiRS7, vB_SepiS_phiIPLA5 and CNPH82-like contigs could be identified in samples 7F, 7S, 10F and 10S, the often high (up to 9000-fold) and even coverage of the Stb20-like contigs suggests that this is not the case. It would further require that for the majority of them, the same phages (CNPH82, vB_SepiS_phiIPLA5) be present in all samples which is unlikely. We therefore consider these as genuine genome reconstructions. This arrangement, of conservation of the physical structural module and variability in the DNA metabolism/lysis/lysogeny modules has been described for the φPLPE group of dwarf-myoviruses, which display a remarkably wide host range^45^. This leaves the question open of whether these phages infect the same genus of bacteria. It has been suggested that generally phage module swapping happens at the level of gene-clusters rather than as individual genes^46^. This clustering approach may however miss nuanced recombination events related to an individual phage or a small group of phages, which might happen at the single gene level. As the proteins in the “back halves” of some of these genomes show highest homology to both CNPH82 or vB_SepiS_phiIPLA5, these recombinations may have happened at the single gene level.

BLASTn of the Stb20 genome sequence against the NCBInr database (February 15th, 2017) show substantial regions of nucleotide identity (55% of the genome at 89% identity) covering the structural genes, to portions of three *S*. *epidermidis* genomes from Finland (HG813242.1), Australia (LT571449.1^47^ and Russia (KT429161.1; 2015), as well as one *Staphylococcus xylosus* genome (LN554884.1)^48,49^. This suggests that these phages are closely associated with *Staphylococcus* species from human skin. These observations suggest three distinct possibilities: 1) That these phages perhaps infect hosts other than *S*. *capitis* and could suggest a wider host range 2) That genetic exchange between phages capable of infecting *S*. *capitis* and other *Staphylococcus* species has resulted in these hybrids or 3) That the various *Staphylococcus* infecting phages that seem to compose the Stb20-like phages shared a common ancestor.

It was demonstrated, in the publication describing Stb20^43^, that its proteins belonged to a unique, new cluster of proteins (“phages”), separate from those of Stb12 and Stb27. Stb20-like phages have not yet been classified into a *Staphylococcus* phage genus and are currently part of an orphan group including Stb12, Stb27, TEM123, 2638 A and SpaA1. In a proposed classification of *Staphylococcus* phages, Gutiérrez and co-workers suggest that Stb20 seems to be distinct from Stb12 and Stb27^50^. The region containing genes related to DNA metabolism/lysis/lysogeny shows some homology between Stb20 and Stb12, but not Stb27. It is also evident that the structural genes of Stb12 and Stb27 are more similar to each other than those of Stb20. Here we found a collection of phages from skin associated bacteria that show strong conservation of the genes required for the physical structure of the phage particle, with variable regions for DNA metabolism/lysis/lysogeny and replication. It may well be that the conserved module is so successful and omnipresent because it has the ability to infect a wide range of bacteria (broad host range), whereas other *Staphylococcus* infecting phages are more species specific and this is reflected in the unique nature of their physical structure, genomic sequence and arrangement. This would argue that this module, and therefore this physical structure, is highly successful in the environment it finds itself, and is likely responsible for the wide host range. Although the authors who described the Stb-phages assayed the host range of Stb12, Stb27 and Stb20 using a variety of *Staphylococcus* species including *S*. *aureus*, they could not confirm a broad-host-range for these phages^43^. The variety of lysis/lysogeny and DNA metabolism modules observed suggests that this region is often modulated or swapped out and is selected for on the basis of timing of these events in a particular host. Thus, the intracellular conditions could select for these modules (variable module), just as the cell wall structures select for the physical structure (conserved module) of the phage. The variation in these modules may lead to different modes of integration, replication and triggers/regulation of lytic induction. Although the evolution of phages through module swapping has been described, the phages described here serve as a particularly good example displaying qualitative mosaicism as defined by Casjens^51^.

The largest contig (>141 kb) in sample 7AX, and the largest genome recovered from the metavirome, was highly similar (BLASTn 89% query coverage/96% identity) to the *Staphylococcus* phages phiIPLA-C1C (Sewage, Spain) and phiIBB-SEP1 (Sewage, Portugal; Supplementary Fig. S3). Studies showed that phage phiIPLA-C1C was capable of efficient lysis of mixed staphylococcal biofilms and both these phages were described as possibly useful in phage therapy^52,53^. While phiIPLA-C1C showed a very broad host range, phiIBB-SEP1 only infects *S*. *epidermidis*^52^. This leaves the question open of whether the phage identified in 7AX has a similarly narrow host range. The 7AX_1 endolysin is disrupted by an endonuclease-like protein unlike the other two phages. The endonuclease appears to be absent in phiIPLA-C1C, and in phiIBB-SEP1 the same endonuclease lies directly upstream of the endolysin and downstream of the holin. This possibly indicates different strategies/timings for host lysis although the mechanisms may be conserved. An antirepressor protein, usually associated with control of the switch between lysis and lysogeny, could be identified on phiIBB-SEP1; however, it is not present on the genomes of phiIPLA-C1C and 7AX_1. This suggests either that this *Sep1virus*, normally considered as purely lytic, can be lysogenic or hints at being previously capable of lysogenizing its host. Similar to phiIPLA-C1C and phiIBB-SEP1, no tRNAs could be identified on 7AX_1 adding another example of this unusual missing feature to the growing *Sep1virus* genus. Five group I introns could also be identified on 7AX_1. One of the two putative helicases encoded by these three viruses is longer in phiIPLA-C1C and 7AX_1 than in phiIBB-SEP1, with the longer versions containing an intein. This is in addition to those initially described for phiIPLA-C1C^53^. The largest differences between these three phages are in the DNA replication, modification and repair regions. Interestingly, the SceD-like transglycosylase encoded by these phages has been proposed as a biomarker for vancomycin intermediate *Staphylococcus aureus* strains^54^. However, should these phages prove to be purely lytic, their usefulness as a biomarker is diminished.

The identification of the 7AX_1 phage in bacteria from a skin sample of the South African cohort, shows that these phages naturally occur with/in bacteria on the skin and is the first time the phage is directly linked with humans, and further hints at their global distribution. If the 7AX_1 phage is purely lytic, as suggested by its genomic content and relation to phiIPLA-C1C, then it should have been present as phage particles on the skin of the sampled individual prior to enrichment. This implies that these phages could already provide some low-level resistance to infection to those individuals who carry the phage-host pair. The phage could not be identified, with confidence, in any of the other samples using read mapping and according to Hannigan *et al*., the majority (>85%) of the phages identified in their skin samples were temperate, which makes the lytic phage 7AX_1 identified here rather rare.

### Pseudomonas phages

One phage which was identified in samples from all individuals is most closely related (80% nucleotide identity over 19% of the length of the genome) to prophages identified on *Pseudomonas savastanoi* NCPPB 3335 genome (CP008742), and more distantly to *Pseudomonas* infecting phage vB_PaeP_Tr60_Ab31. Remarkably, the sequence of all contigs identified was perfectly conserved at the nucleotide level. One possible explanation is that these sequences were derived from contamination of the samples, in which case one could expect them to be present in all samples, which was not the case. Furthermore, it could be identified in other skin metagenomic datasets (see below). This may point to the host being ubiquitous in the environment and often shared between individuals, resulting in the same sequence being detected, or that strong selective pressure exists to maintain the genome as is. Given what is known of phage evolution, the latter option seems less likely^55^. However, in the skin environment, one exception appears to be *Propionibacterium* phages^20^. The vB_PaeP_Tr60_Ab31-like phages described here could be another one. According to Hannigan *et al*., the *Propionibacterium* phages are under reduced pressure to adapt, as reflected by their very similar genomes. In our view, evolutionary pressure always exists, and the occurrence of such similar genome sequences may, in agreement with the Hannigan findings, more likely be the result of a restricted host range (selective pressure) rather than reduced evolutionary pressure.

vB_PaeP_Tr60_Ab31 was described as a chimera between siphovirus PAJU2 and podovirus AF^56^. The genomes described here partially share the genome organization of vB_PaeP_Tr60_Ab31 (Supplementary Fig. S4); however, amino acid similarity to the vB_PaeP_Tr60_Ab31 phage is very low. Thus, even though the genome arrangement is conserved, the amino acid sequences are not. Thus, this would suggest that these genomes represent a similar but separate occurrence of rearrangement between phages to create an Ab31-like genome arrangement. It appears that two regions on these genomes (head and tail morphogenesis) are most similar to that of a prophage in *Pseudomonas putida* strain PC2 (CP011789.1). The region involved with DNA replication, repair and modification, which includes the tail spike protein, acyltransferase and integrase is however more closely related to a podovirus-like prophage found in *Pseudomonas fluorescens* (LCYD01000004.1^42,57^). It is interesting to note that these Ab31-like phages and the *P*. *fluorescens* prophage encode acyltransferases are all bracketed by an integrase and tail spike protein. The role of these enzymes when present on phage genomes has not often been investigated^58,59^. In the case of phage LUZ19, that infects *P*. *aeruginosa*, it was found that expression of the acyltransferase is responsible for a significant reduction in host transcription (30%) and replication during infection. In contrast, the acyltransferase found on prophage SfII in *Shigella flexneri*, is responsible for serotype conversion. What role the acyltransferase might play in this group of Ab31-like phages has yet to be determined. The fact that overall, these genomes appear most closely related to prophages, likely indicates that they too are temperate. No easily identifiable terminase could be found on any of these genomes, nor on the prophage that it seems most closely related to. No other contig of equal coverage, potentially harbouring the terminase, could be identified in these same samples. The high coverage of some of these genomes together with the recovery of the same contig length from several individual samples (which were circularly permuted relative to each other; 37851 bp contig ex 9 S detected as circular) suggests that these genomes are complete, or nearly so. It may be that the terminase, in this case, is radically different from those found in current databases or this role may be fulfilled by other terminases present in the host analogous to the ΦAH14a and ΦAH14b satellite phage helper system^60^.

The highly novel, narrow host range, siphovirus phiPSA1 that infects *Pseudomonas syringae* was shown to be temperate^61^. Twenty-seven contigs showed best BLAST hit to phiPSA1 (Supplementary Table S2) and as for this phage, they show highest similarity to prophages on a wide variety of *Pseudomonas* genomes, all of which encode integrases indicating that they are likely temperate. These phiPSA1-like phages identified in the various samples were characterized into two groups. For one group with genomes over 53 kb (>53 kb group), there was only one nucleotide difference in their nucleotide sequence, while for the second group with a genome size of ±49 kb (one detected as circular 49209 bp), there were 2 base changes over the length of the “full genomes”. As for the Ab31-like phages described above, these findings show that identical phages (and their hosts) can occur on the skin of unrelated individuals, and in different body sites. Here we compare genomes representative of these two groups to prophage relatives from *P*. *putida* JB (CP016212), *Pseudomonas moraviensis* BS3668 (LT629788) and phiPSA1 (Supplementary Fig. S5). Compared with their respective prophage relatives, both groups ( ±49 kb and >53 kb) share high similarity in the regions coding for the physical structure of the phage, apart from a much longer tail fiber-like protein in the prophage from BS3668. In contrast, the ±49 kb phages shared amino acid similarity to the phiPSA1 head proteins while the >53 kb genomes shared similarity over the proteins which make up the phiPSA1 tail region. The vConTACT analysis also indicated that the phages with taxonomic affiliation to Enterobacteria phage mEp235 shared protein clusters with the ±49 kb phiPSA1-like genomes (Fig. 2A).

A Mu-like phage found in a variety of samples (Supplementary Table S2) showed highest nucleotide identity to a prophage of *P*. *putida* S13.1.2 (CP010979.1). All the genomes identified across a range of body sites and individuals were 100% identical. Best BLAST analysis showed that several *Pseudomonas* JBD phages, as well as *Mannheimia* phage vB_MhM_3927AP2 and *Vibrio* phage martha12B12 were distant relatives to those identified in our study (Supplementary Fig. S6). A unique feature of the JBD phages is an anti-CRISPR gene cassette which protects the phage against degradation^62^. However, no such cassette could be identified on the genomes described here. Despite the absence of such a cassette, no CRISPR spacer against this phage could be identified (see below). Another feature shared by four of the genomes compared here is a DNA circularization protein located in amongst the tail components, previously shown to play multiple roles in the Mu “reproductive cycle”. It appears to be an integral part of the tail itself, protecting the phage genome from host endonucleases after injection and is responsible for circularizing the phage DNA once injected into the cell^63^. No such protein could be identified on the JBD25 and JBD69 genomes. Although the genomes compared here show low nucleotide and amino acid identity, the genome organization and protein functions, especially the lysis, head and tail component regions are highly conserved as previously noted for Mu-like phages^64^. The DNA replication modification and repair region of JBD25 also stands out compared with the other phages, seemingly having undergone several recombination events resulting in truncation of the transposase and loss of the associated transposition protein as well as the acquisition of an integrase, not present or easily identified in the other genomes. It also features an extended lysis module. JBD69 has been shown to form lysogens, and the *Pseudomonas* S13.1.2 prophage obviously also does, indicating that an integrase should be present on these and the Mu-like phages identified here^65^. As a well-known generalized transducing phage, Mu-like phages could potentially play a role in helping species to adapt to various environments through horizontal transfer and could perform the same function on the skin for the hosts they infect^66^. A phage with relation to *Pseudomonas* infecting cytotoxin-converting phage phiCTX was also identified in sample 3S^67^, however no *ctx* toxin could identified on the genome (Supplementary Fig. S7).

### Acinetobacter phages

Two of the phage genomes that were detected as circular from sample 2F (2F_1 and 2F_2; Supplementary Table S2) likely originate from *Acinetobacter* species. The host assignment is based on high nucleotide identity for segments of the phage genomes (BLASTn 34% query coverage/77% identity for 2F_1 and BLASTn 5% query coverage/85% identity for 2F_2) as well as high amino acid identity of many of the proteins encoded by these phages to *Acinetobacter* genomes on GenBank, suggesting that there are prophage relatives on these genomes. However, these were not identified as possible hosts by purely looking at the taxonomic assignment given to contigs, showing the current limitations of viral metagenome analysis for both these phages, a variety of proteins of which share highest protein similarity to a range of prophage genomes as opposed to just one (Supplementary Fig. S8). 2F_2 is most similar to prophages from *Acinetobacter junii* SH205 (GG705011.1; isolated from skin) and *Acinetobacter baumannii* strain A1 (CP010781). It is more distantly related to *Acinetobacter* phages LZ35, YMC-13-01-C62 and *Enterobacteria* phage BP-4795. The smaller of the two (2F_1) is also related to prophages on many *Acinetobacter* genomes, and here we compare it to prophage genomes from *Acinetobacter* sp. TGL-Y2 (CP015110) and *Acinetobacter johnsonii* ANC 3681 (APPZ01000010). The possible recombination between multiple phages is illustrated by (as an example) the terminase having highest similarity to a protein from *A*. *johnsonii* while the portal protein is most similar to one from *A*. *junii*.

Phage 2F_1 also shows some similarity to the *Burkholderia* infecting phage KS14. A unique feature of phage KS14 is that it is maintained as a plasmid^68^. Whether the phage from 2F is also maintained as such needs to be determined. Unlike KS14 and related phage P2, the 2F_1 phage does not appear to have a five-gene lysis module. Instead, only one protein could be assigned a function related to cell lysis, ORF9, which encodes a putative glycoside hydrolase. It is expected that ORF8 which lies directly upstream of ORF9 and has three predicted transmembrane regions encodes the holin, even though it shows no homology to known holins. At least two proteins (topoisomerase and tape measure protein) on the prophage genome found in *Acinetobacter* TGL-Y2 appear to be produced through transcriptional slippage, which does not appear to be the case for the phage from 2F (Supplementary Fig. S8). A DNA adenine methyltransferase is present on the genomes of the 2F phage as well as the *Acinetobacter* prophage between ORFs 11 and 13, and although a DNA modification function can easily be assigned to it, its absence from the KS14 genome, the *A*. *johnsonii* prophage and its location in amongst the tail genes suggests that it is a recent acquisition and may represent a moron position. This phage, being P2-like in terms of its genome layout but not in terms of amino acid sequence similarity, could tentatively be classed as belonging to the *Peduovirinae* subfamily.

Although not shared as widely as many of the other phages described here, an identical 40 kb phage was identified in the axilla and forearm samples of two individuals (7F and 7AX, and 8AX). The taxonomic affiliation assigned by MetaVir is *Burkholderia* phage Bcep176. However, given the high amino acid sequence identity of most phage encoded proteins to prophage proteins on *Acinetobacter* genomes, it is more likely that this phage infects *Acinetobacter* species.

The identification of phages related to those present on many *Acinetobacter* genomes suggests a wide spread infection of these bacteria by such viruses. *Acinetobacter* species have emerged in recent years as a significant threat to human health^69^ and phage therapies are sought that could be employed to target these pathogens. The identification here of two novel phages putatively targeting these microbes adds to the list of phages that could potentially be used to combat such infections.

### Presence of skin phages in other metagenomic datasets

To determine whether the phages described here were unique to our dataset or if they could be identified in other publicly available skin metagenome and metavirome datasets, reads and contigs from four select skin metagenome and metavirome studies were mapped against our phage genomes (Supplementary Table S3). For the Oh and Human Microbiome Project (HMP) datasets, only retro auricular crease (behind the auricle of the ear) data was interrogated. Reads from the total Hannigan dataset could be mapped to 130 of our contigs and to 29 from the HMP retro auricular crease data (Supplementary Table S3). Of the unique reads mapped to our contigs, the bulk mapped to *Staphylococcus* phage-like contigs with 79% from the HMP data and 61% from the Hannigan data. Non*-Staphylococcus* phage-like contigs only recruited a small number of reads each from these datasets (Supplementary Table S3) with two exceptions; the phiCTX-like phage and a phiSM-like *Cellulophaga* phage from subjects 3 and 9 also present in the Hannigan dataset.

Although large portions of the Stb20-like genomes could be reconstructed from the Hannigan dataset, and to a lesser degree the HMP data, they could not be identified in the Chng or Oh datasets (Supplementary Table S3). They were the group of phages that recruited most reads from both these datasets with 54% of unique mapped reads from the HMP data and 23% from the Hannigan data. The 12562 reads that mapped to the 16872 bp contig and 6705 reads that mapped to the 13110 bp contig, both from sample 7S, were not considered as genuine, as the bulk of these reads were small (>45 bp or >80 bp) and mapped to only one location on either contig. Only one of our contigs could tentatively be identified in the Oh data, the *Staphylococcus* infecting IME_SA4-like phage, with one 200 bp read mapping to its terminase large subunit (3S, 42828 bp). Given the evidence of a global distribution for Stb20-like phages in our study and its substantial presence in the Hannigan dataset, where skin viruses were targeted, it is surprising not to find them in the Oh or Chng datasets. However, differences in participant demographics and/or health status could account for this. The Hannigan and Oh datasets were produced from North American volunteers, while the Chng data was from Asian volunteers. It has been demonstrated that the Chinese community has a different microbial community composition compared to Western individuals, with lower relative abundance of *Staphylococcus* species^70,71^. Furthermore, the Chng study data was generated from individuals with atopic dermatitis, a condition that may result in an altered skin microbiome and therefore phage community. These two factors may explain the absence of these phage signatures. It appears that the technique (metavirome vs metagenome) used to specifically probe for skin viruses (Hannigan study) resulted in a dataset from which we could map reads to the greatest number of our contigs. It may be expected that there could be differences between the Chng and Oh/Hannigan data, however, our inability to detect even traces of these phages in these datasets, either indicates their absence, or that deeper sequencing or targeted sampling is required to detect them.

To establish whether the phages identified here were unique to skin or are readily found in other environments, we compared our contigs to those in the “earth virome” contig set^72^. No contigs larger than 10 kb found a full-length match in the “earth virome” contig set indicating the novelty of the phages identified here (Supplementary Table S3). We could however detect the presence of related phages. Of the unique phages, large portions of three could find matches in this dataset. The phage with the closest match to a contig in the “earth virome” was phiILPA-C1C with >108 kb of its genome recovered at 100% nucleotide identity, also from a human skin metagenome sample. Large portions of *Streptococcus* 20617-like phage (63% of the genome) and *Vibrio* VP882-like phage (65% of the genome) from 3S could also find sequence similarity.

**Figure 4:**
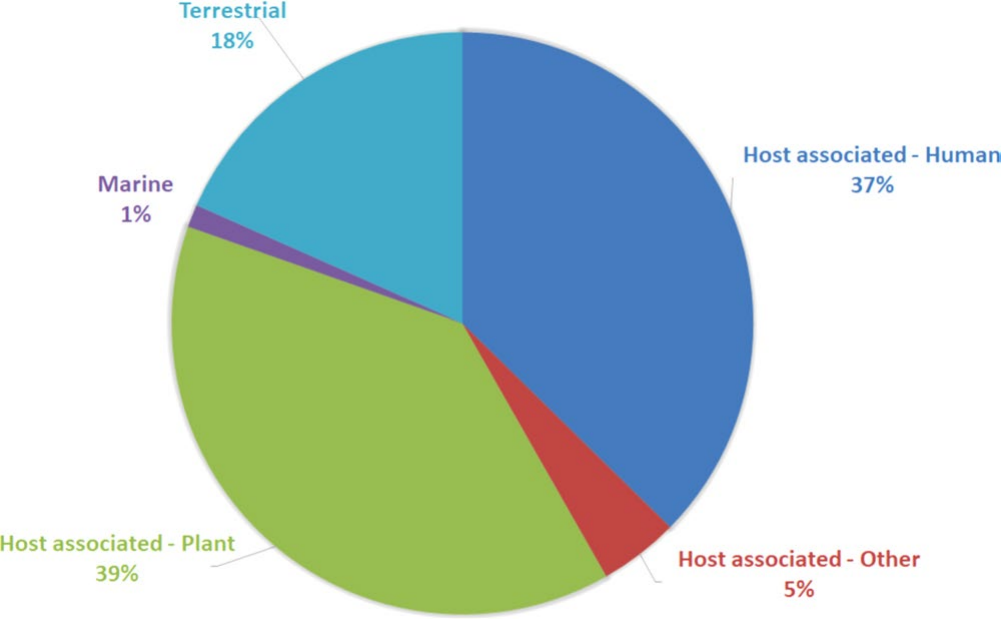
BLASTn analysis of the 159 contigs identified here against the “earth’s virome” dataset showing the association of each contig with an environmental niche.

37% of the top hits were related to HMP data (skin, nares, stool or tongue dorsum), suggesting that these phages are most closely associated with human samples (Fig. 4; Supplementary Table S3). The remainder (63%) have best BLAST hit to other sources indicating that many of these contigs could come from sources other than the skin microbes themselves. Whether this resulted from the acquisition of the phages from the environment on the day of sampling or due to a database bias toward environmental samples cannot be discerned. 97% of the *Staphylococcal*-phage hits were to sequences derived from human samples and for the Stb20-like contigs, most hits were to retro auricular crease data, and mostly absent from environmental datasets. Of the unique contigs, 55% were human associated and 22% of shared phages. Most of the best hits for *Bacillus*- and *Pseudomonas*-like phages were to contigs from soil (rhizosphere) communities.

Interestingly, two of our contigs showed highest nucleotide identity to contigs from a wastewater bioreactor metagenome study conducted in Cape Town (Gs0118793). One contig has highest taxonomic affiliation with *Enterobacteria* phage HK446 and the other to *Salmonella* phage FSLSP-058. In both cases, the longest hit was over 2.6 kb at >88% nucleotide identity. Such a biogeographical link could indicate that these phages and their hosts are transient members of the skin community and that their presence is of environmental origin.

### CRISPR signatures

The Hannigan study demonstrated that CRISPR spacers targeting certain phages were present in one area of the body and absent in others, and that this could partly explain the unique compositions of the phage communities. We therefore examined whether there are CRISPR “adaptive immunity” systems targeting the phages described here by BLASTn comparison of the genomes identified here against the CRISPR finder database. Spacers could tentatively be identified for 115 of the 159 contigs. For many, the organism in which potential spacer hits were found are evolutionarily distant from the proposed host identified in our analysis (Supplementary Table S4). Only 34 of the 115 identified contigs had spacers in genera that correlated with the taxonomic assignment, and these were mostly dominated by *Pseudomonas*-infecting related phages. For the Ab31-like *Pseudomonas* phages a spacer (32 nt) was identified with two nucleotide mismatches in the recently sequenced (2015) *Pseudomonas* sp. MRSN12121 GCF_000931465, a multidrug resistant isolate from urine. For the two groups of phiPSA1-like phages, five unique spacers were found. For the ±49 kb group, two spacers of 30 nt with one mismatch and one of 29 nt with two mismatches were found in another recently sequenced (2016) isolate *Pseudomonas chlororaphis* GCF_001602135 isolated from soil. For the >53 kb group, three spacers, of which one had 100% nucleotide identity, was identified in *Pseudomonas parafulva*.

Of the *Staphylococcus*-infecting phages identified, the phiETA3-like, 2638A-like, and vB_SepiS_phiIPLA5-like contigs had spacers directed against them. These were identified in *S*. *aureus* and the coagulase variable species *Staphylococcus schleiferi* and *Staphylococcus agnetis*. The spacer against vB_SepiS_phiIPLA5-like had a 100% match in the genome of *S*. *schleiferi*. The spacer regions identified for the 15604 bp and 9665 bp contigs from 7F, which were assigned a taxonomic affiliation of *Lactobacillus* phage A2, were identified in both *Staphylococcus* and *Lactobacillus* species, with one of them being shared with the 40473 bp *Staphylococcus* 2638A-like phage contig also from 7F. The spacer identified in *Lactobacillus* is shared with the 43445 bp contig from 10S where that phage is also easily identified as one infecting a *Staphylococcus* species. It could be that the taxonomic affiliation is incorrect, since a BLASTn of the contig showed high nucleotide identity with segments from *Staphylococcus* genomes (Supplementary Table S2). Thus, although the taxonomic affiliation and spacer was found in *Lactobacillus*, we think this is coincidental. These two contigs share nucleotide similarity over a 4.6 kb segment at 82% identity, in which the spacer is located.

No spacer regions against Stb20 or our Stb20-like phages could be identified in CRISPR arrays from *Staphylococcal* genomes. Spacers with three base mismatches could however be identified in *Methanosarcina lacustris*, two *Thermoanaerobacter* sp. X513 CRISPR arrays and in the *Caldicellulosiruptor saccharolyticus* DSM 8903 genome. These results are not expected to be a true reflection of the phages’ host range, and warns about the use of CRISPR spacer detection for host range determination. The absence of CRISPR spacers targeting this phage is surprising given its apparent ubiquity in genomes. This absence may be a database limitation, although *Staphylococcus* genomes are some of the best represented in current databases, or could point to a mechanism of CRISPR evasion or selection against protospacer sequences employed by these phages to avoid CRISPR mediated destruction, which has not yet been described.

## Conclusion

This study describes an enriched human skin metavirome that shows similar phage signatures to the only other dataset dedicated to studying human skin virus populations. A caveat of our analysis is that as it is an enriched metavirome, and therefore is by no means a complete representation of the skin virome. It does not reflect which phages are present on the skin at a particular point in time but gives insight into which phages are present in skin-associated bacteria. Our study does highlight one of the limitations associated with current metavirome analysis. We show two examples of hybrid phages (Stb20-like and Ab31-like) which, as smaller contigs in a metavirome study, could be assigned different taxonomies based on sequence similarity. This begs the question as to how many such fragments are present in current metavirome datasets artificially increasing the diversity of phages present when using reference based taxonomic assignments. The solution, where whole phage genomes can be reconstructed, may be to compare contigs across metagenomic datasets and first assign all contigs with similar (identical) sequence to one taxonomic affiliation. A long k-mer based analysis such as that used by the crAss program can be useful to develop a better understanding of the variability between individuals and body sites or metaviromes of any study site^73^.

Despite most studies indicating that the microbial composition is unique per person and per body site, the identification of several phages from our dataset in those generated in geographically distant locations seems to support the notion that although the microbiomes are overall unique, there are clearly shared bacteria and viruses as noted previously^72^. The question is whether these are identical or more distantly related. This can only be answered with the recovery of full length genomes. Furthermore, the presence of the phiPSA1-like and Ab31-like phages described here seems to show that highly similar, if not identical, phages may be in operation on the skin of people who live in close geographical proximity. This should be expected in urban settings where the exchange of skin microbes may be a frequent occurrence. If identical or nearly identical phages are found to be universally present on healthy skin, but not on “diseased” skin, they could be explored as biomarkers for detecting imbalances in skin microbial communities.

If we consider our BLASTn results as a better predictor of the viral host for the various contigs described here, then many of them infect species, the genomes of which *are* adequately represented on the NCBInr database, from where the CRISPR database derives. This would suggest that for the majority of phages identified here, there are currently no CRISPR systems targeting them, and would indicate that there is no limitation for these to spread from one body site to another due to CRISPR-based exclusion and points to other reasons for the exclusion of certain phages from a particular body site. Recent studies which highlight the nuanced behavior of phages even in well studied systems such as that of phage T4 and λ, shows the need to explore phage-host interactions at the molecular level to gain a more complete understanding of these interactions^74–78^. It is possible that phages hold the key to understanding bacterial community imbalances and resultant disorders. These phages undoubtedly have an impact on their host population through lysis events or altered metabolism during lysogeny. However, the implications of this for certain skin disorders in which *Staphylococcus*, *Pseudomonas* or *Acinetobacter* species play a pivotal role, if any, has yet to be determined.

## Electronic supplementary material

Supplementary figures

Table S1

Table S2

Table S3

Table S4
