# Supplementary material for: Novel phages of healthy skin metaviromes from South Africa
Source: Sci Rep. 2018 Aug 16;8:12265. doi: 10.1038/s41598-018-30705-1 (PMC6095929; doi:10.1038/s41598-018-30705-1)
Supplement: Supplementary file 1 — Supplementary figures [file 41598_2018_30705_MOESM1_ESM.pdf]

Title: Novel phages of healthy skin metaviromes from South Africa

Running title: Phages from skin metavirome

Leonardo Joaquim van Zyl[1]*, Yoonus Abrahams[1], Emily Amor Stander[1], Bronwyn Michelle Kirby-McCollough[1], Roland Jourdain[2], Cécile Clavaud[2], Lionel Breton[2], Marla Trindade[1]

[1] Institute for Microbial Biotechnology and Metagenomics (IMBM), University of the Western Cape, Robert Sobukwe Road, Bellville, Cape Town, South Africa.

[2] L'Oréal Research and Innovation, 1 Avenue Eugène Schueller, 93600 Aulnay sous Bois, France

* Author to whom correspondence should be addressed; Leonardo Joaquim van Zyl, e-mail: vanzyllj@gmail.com; Tel.: +27 21 959 2325

Email: Leonardo Joaquim van Zyl: vanzyllj@gmail.com; Cécile Clavaud: cclavaud@rd.loreal.com; Roland Jourdain: rjourdain@rd.loreal.com; Lionel Breton: lbreton@rd.loreal.com; Marla I. Trindade: prof.marlatt@gmail.com

Keywords: Bacteriophage, skin, Stb20, metagenomics

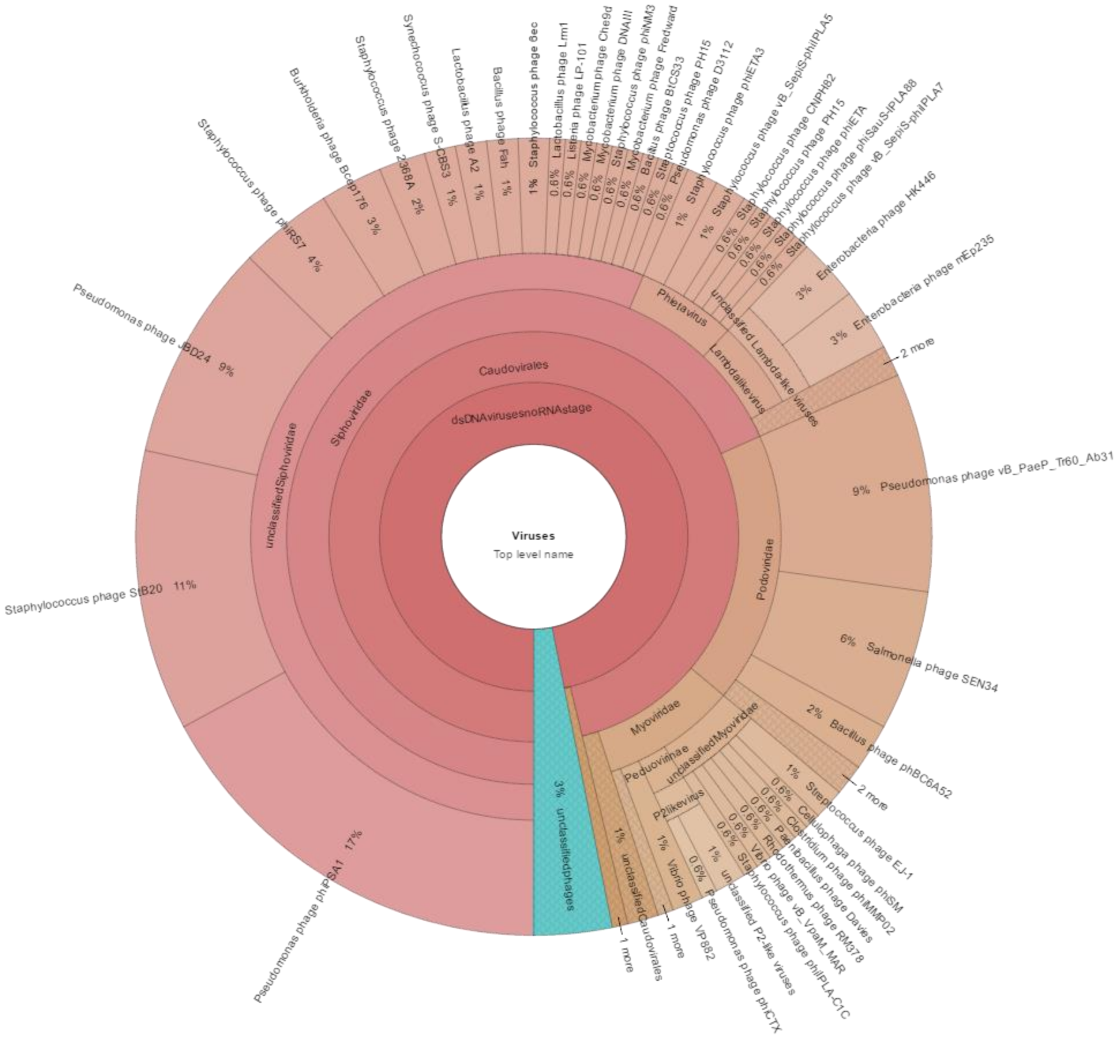

Figure S1. Taxonomic profile of contigs recovered from the combined skin metaviromes

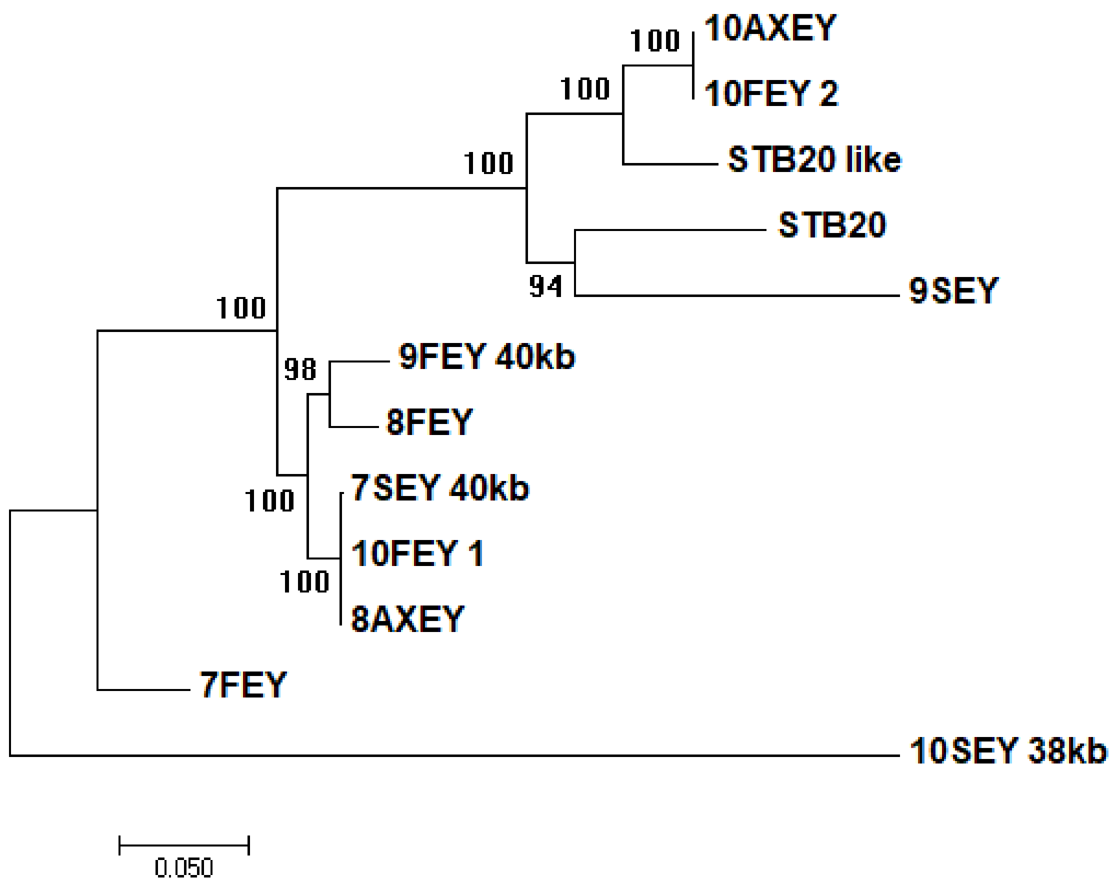

Figure S2. The evolutionary history of Stb20-like phages was inferred using the Neighbor-Joining method. Branch lengths in the same units as those of the evolutionary distances used to infer the phylogenetic tree.

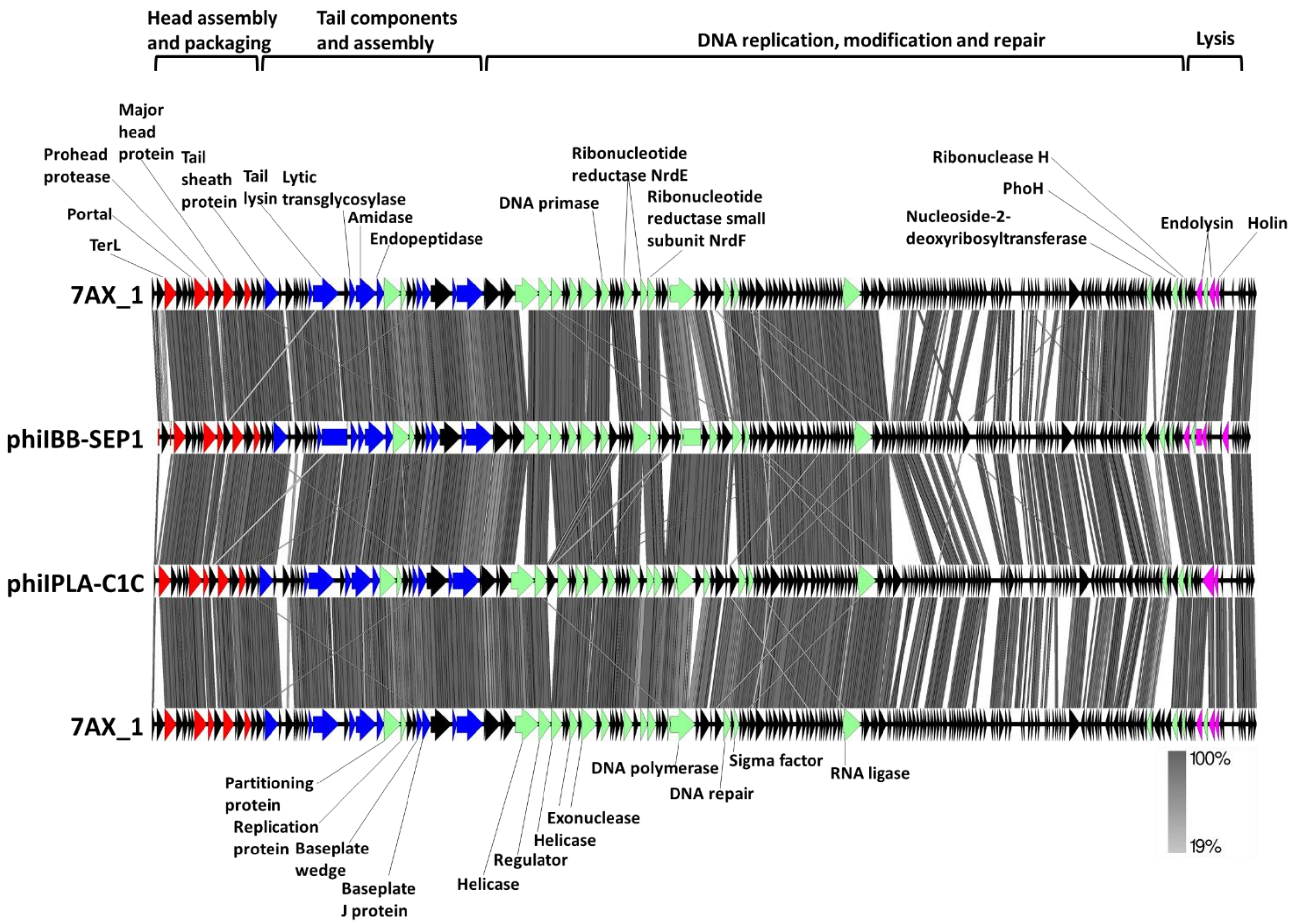

Figure S3. Comparison of the genomic structure of phiIPLA-C1C, phiIBB-SEP1 and a related phage from 7AX

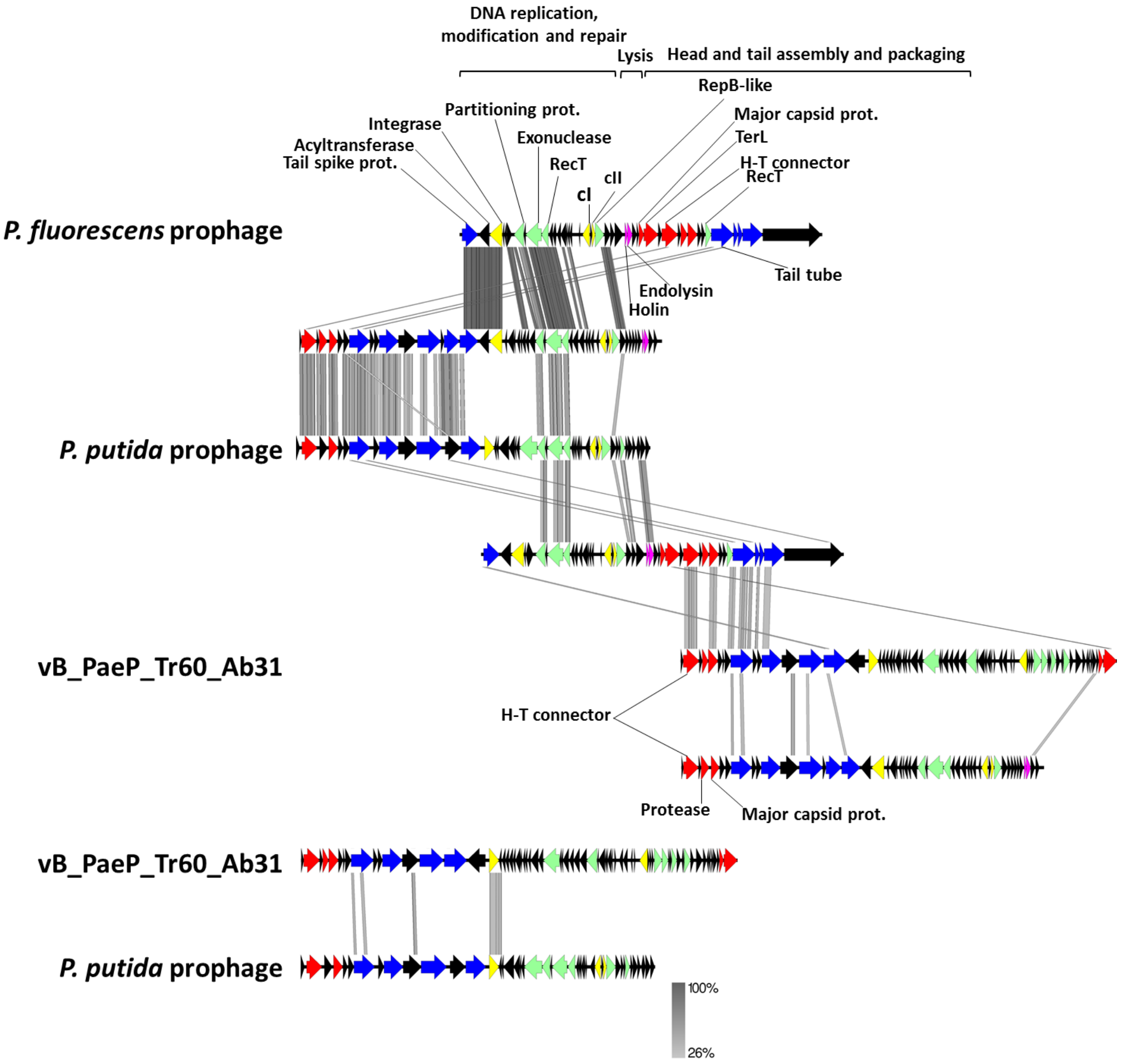

Figure S4. Comparison of the genomic structure of Ab31-like phages from 2AX, 3S, 7F, 7S, 7AX, 8AX, 8S, 9S, 9AX, 10F, 10AX to their closest relatives

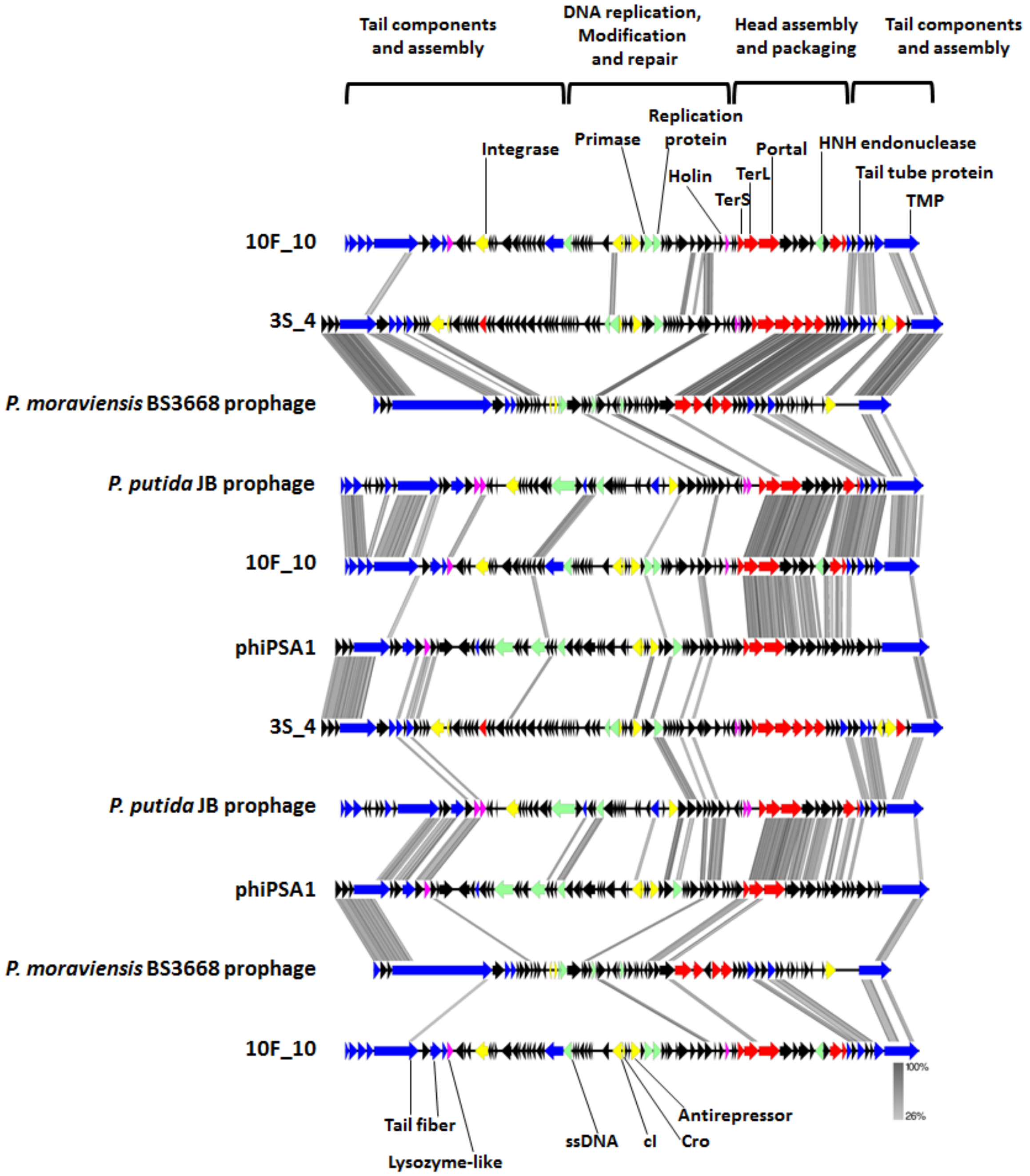

Figure S5. Comparison of the genomic structure of phiPSA1-like contigs from 10F and 8S to its closest relatives

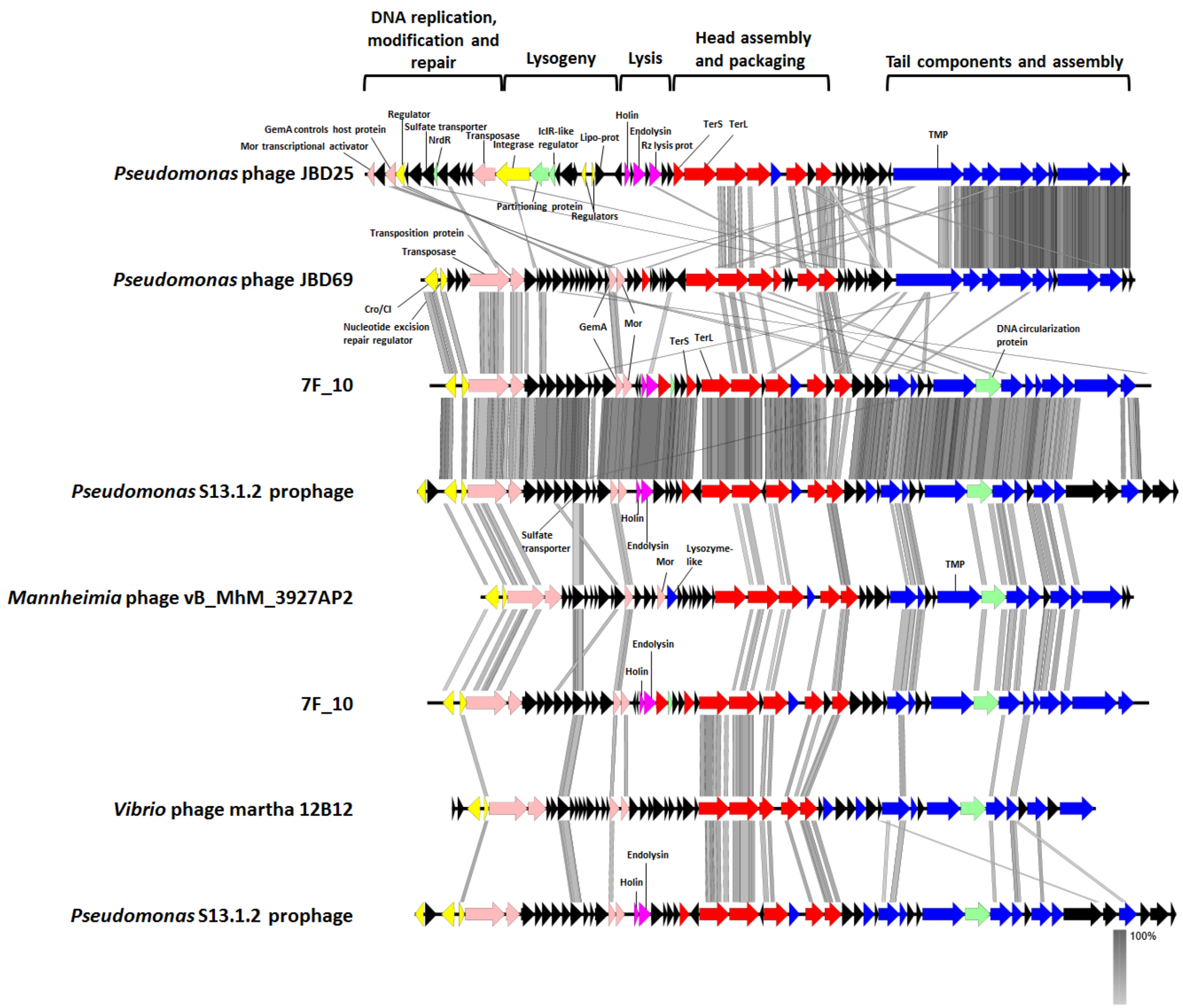

Figure S6. Comparison of the genomic structure of *Pseudomonas* infecting Mu-like phages

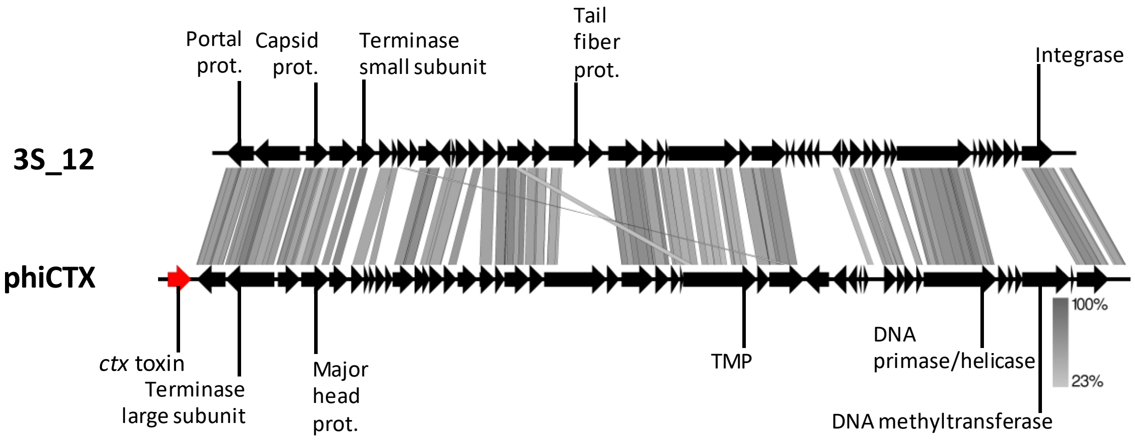

Figure S7. Comparison of the genomic structure of phiCTX-like contig from 3S and phiCTX. The ORF highlighted in red is the *ctx* toxin

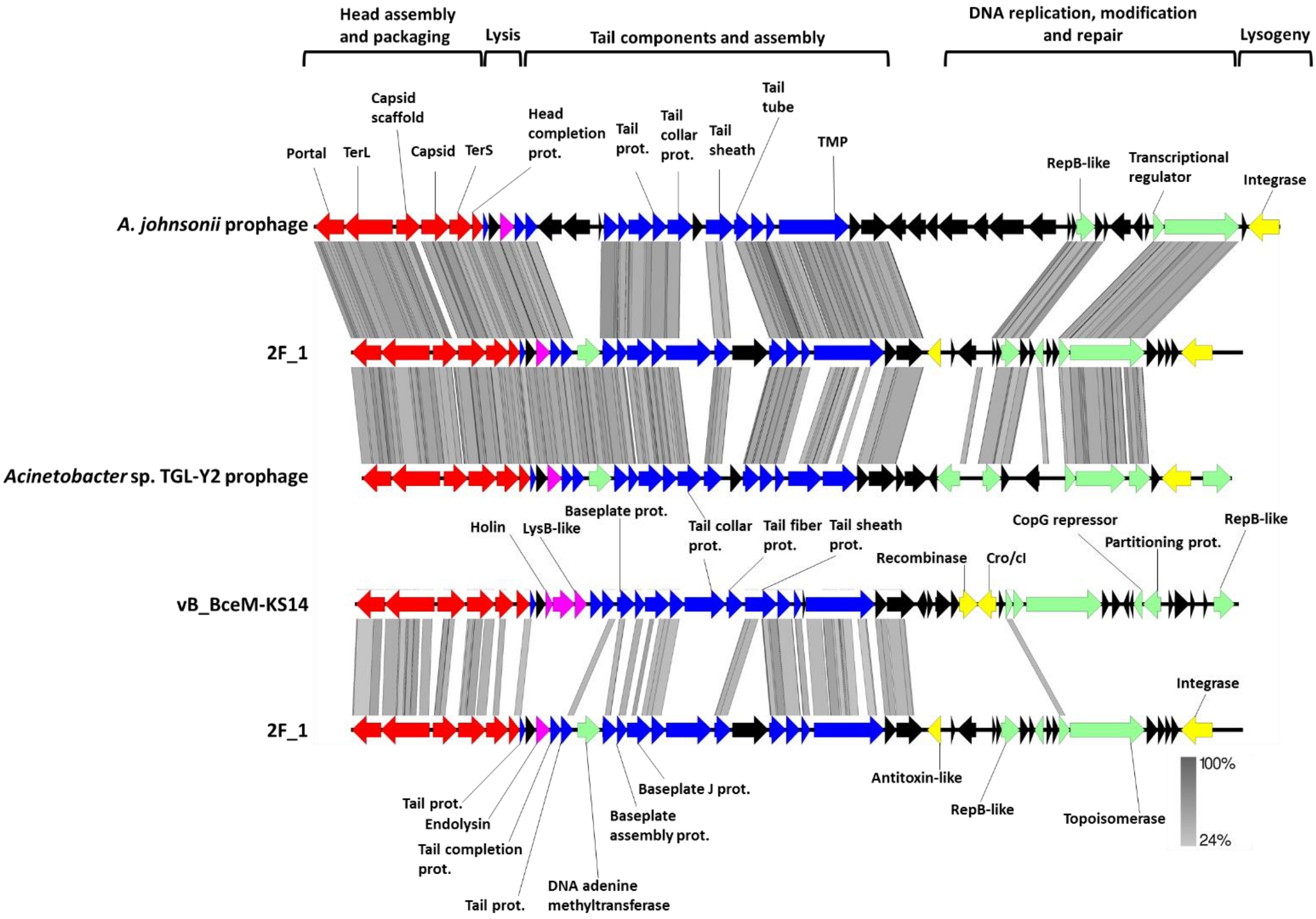

Figure S8. Comparison of the genomic structure of the vB_Bcem-KS14-like contig from 2F to its closest relatives
