# Supplementary material for: Novel phages of healthy skin metaviromes from South Africa
Source: Sci Rep. 2018 Aug 16;8:12265. doi: 10.1038/s41598-018-30705-1 (PMC6095929; doi:10.1038/s41598-018-30705-1)
Supplement: Supplementary file 5 — Table S4 [file 41598_2018_30705_MOESM5_ESM.docx]

| **Sample** | **Contig size** | **Taxonomic affiliation** | **CRISPR Host** | **CRISPR spacer sequence match** |
| --- | --- | --- | --- | --- |
| 10S | 6187 | *Salmonella* phage FSLSP-058 | *Thermanaerovibrio velox* | ccgccgcgcgctggcgtggat  \|\|\|\|\|\|\|\|\|\|\| \|\|\|\|\|\|\|\|\| ccgccgcgcgcgggcgtggat |
| 7S | 22381 | *Rhodococcus* phage REQ3 | *Lysobacter antibioticus* | tgtcg-cgccggcgccgagtgtcg  \|\|\|\|\| \|\|\|\|\|\|\|\|\|\|\|\|\| \|\|\|\| tgtcgtcgccggcgccgag-gtcg |
| 7F | 20166 | *Staphylococcus* phage phiRS7 | *Synechococcus* sp | tttccaactcaa-ccattccaacg  \|\|\|\|\|\|\|\|\| \|\| \|\|\|\|\|\|\|\|\|\|\| tttccaact-aatccattccaacg |
| 3F | 57508 | *Pseudomonas* phage phiPSA1 | *Pseudomonas fluorescens*  *Pseudomonas fluorescens* | tcgcgagcaagcccgctcc  \|\|\|\|\|\|\|\|\|\|\|\|\|\|\|\|\|\|\| tcgcgagcaagcccgctcc  ggagcgggcttgctcgcga  \|\|\|\|\|\|\|\|\|\|\|\|\|\|\|\|\|\|\| ggagcgggcttgctcgcga |
| 9S | 45052 | *Staphylococcus* phage 6ec | *Leptotrichia* sp  *Sulfolobus islandicus* | gaaca-gaattgattactaaaaatttaa  \|\|\|\|\| \|\|\|\|\|\|\|\|\|\| \|\|\|\|\|\|\|\|\|\|\| gaacaagaattgatta-taaaaatttaa  tttgtcataatatataatata  \|\|\|\|\|\|\|\|\|\|\|\|\|\|\|\|\|\|\|\|\| tttgtcataatatataatata |
| 9S | 41553 | *Staphylococcus* phage StB20 | *Moorella thermoacetica*  *Thermoanaerobacter sp* | aaagaggatttgta-acacctgaagaatatttag  \|\|\| \|\|\|\|\|\|\| \|\| \|\| \|\|\|\|\|\|\|\|\|\|\|\|\|\|\|\| aaa-aggattt-tacactcctgaagaatatttag  atttaggagtg-ttttaatatggcagagaaaaa  \|\|\|\|\|\|\|\|\|\|\| \|\|\|\|\| \|\|\|\|\|\| \|\|\|\|\|\|\| atttaggagtgatttta--atggca-agaaaaa |
| 9S | 37851 | *Pseudomonas* phage Ab31 | *Pseudomonas* sp | cagaactgctcaaacttctccggcttcatgcg  \|\|\|\|\|\|\|\| \|\| \|\|\|\|\|\|\|\|\|\|\|\|\|\|\|\|\|\|\|\| cagaactgttcgaacttctccggcttcatgcg |
| 10AX | 42205 | *Staphylococcus* phage Stb20 | *Moorella thermoacetica*  *Methanosarcina lacustris*  *Thermoanaerobacter sp* | ctaaatattcttcaggcgt-tacaaatcctcttt  \|\|\|\|\|\|\|\|\|\|\|\|\|\|\|\| \|\| \|\| \|\|\|\|\|\|\| \|\|\| ctaaatattcttcaggagtgta-aaatcct-ttt  attcataatttctttgttatctataatcgc  \|\|\| \|\|\| \|\|\| \|\|\|\|\|\|\|\|\|\|\|\|\|\|\|\|\|\| att-ata-ttt-tttgttatctataatcgc  tttttctctgccatattaaaa-cactcctaaat  \|\|\|\|\|\|\| \|\|\|\|\|\| \|\|\|\|\| \|\|\|\|\|\|\|\|\|\|\| tttttct-tgccat--taaaatcactcctaaat |
| 10AX | 38064 | *Pseudomonas* phage Ab31 | *Pseudomonas* sp | cagaactgctcaaacttctccggcttcatgcg  \|\|\|\|\|\|\|\| \|\| \|\|\|\|\|\|\|\|\|\|\|\|\|\|\|\|\|\|\|\| cagaactgttcgaacttctccggcttcatgcg |
| 10AX | 5467 | *Pseudomonas* phage phiPSA1 | *Clostridium stercorarium*  *Corynebacterium ulcerans* | tctgctt-t-tcggcggcggccagggc  \|\|\|\|\|\|\| \| \|\|\|\|\|\|\|\|\|\|\|\|\|\|\|\|\| tctgcttctgtcggcggcggccagggc  aggc-gcgactcgaaagccgcagcga  \|\|\|\| \|\|\|\|\| \|\|\|\| \|\|\|\|\|\|\|\|\|\| aggctgcgac-cgaa-gccgcagcga |
| 10F | 49390 | *Pseudomonas* phage phiPSA1 | *Pseudomonas chlororaphis*  *Clostridium stercorarium*  *Pseudomonas chlororaphis* | ctc-ccccgcgcccatcggcaaccagcggga  \|\|\| \|\|\|\|\|\|\|\|\|\|\|\|\|\|\|\|\|\|\|\|\|\|\|\|\|\|\| ctcaccccgcgcccatcggcaaccagcggga  tctgctt-t-tcggcggcggccagggc  \|\|\|\|\|\|\| \| \|\|\|\|\|\|\|\|\|\|\|\|\|\|\|\|\| tctgcttctgtcggcggcggccagggc  ggcagcatgatcagcaaccacctcagcct  \|\|\|\| \|\|\|\|\|\|\|\|\|\|\|\| \|\|\|\|\|\|\|\|\|\|\| ggcaccatgatcagcaatcacctcagcct |
| 10F | 41090 | *Listeria* phage B054 | *Bacillus pumilus*  *Bacillus pumilus* | ccatacgcctttactgacattgcaaatagcggtaga  \|\|\|\|\|\|\|\|\|\|\|\|\|\| \|\|\|\|\|\|\|\|\|\|\|\|\|\|\|\|\|\|\|\|\| ccatacgcctttacggacattgcaaatagcggtaga  atttcattttcaagcttcacccaatggcagc  \|\|\|\|\|\|\|\|\|\|\|\|\|\|\|\|\|\|\|\|\|\|\|\|\|\|\|\|\|\|\| atttcattttcaagcttcacccaatggcagc |
| 10F | 38959 | *Staphylococcus* phage StB20 | *Moorella thermoacetica*  *Methanosarcina lacustris*  *Thermoanaerobacter* sp | ctaaatattcttcaggcgt-tacaaatcctcttt  \|\|\|\|\|\|\|\|\|\|\|\|\|\|\|\| \|\| \|\| \|\|\|\|\|\|\| \|\|\| ctaaatattcttcaggagtgta-aaatcct-ttt  attcataatttctttgttatctataatcgc  \|\|\| \|\|\| \|\|\| \|\|\|\|\|\|\|\|\|\|\|\|\|\|\|\|\|\| att-ata-ttt-tttgttatctataatcgc  tttttctctgccatattaaaa-cactcctaaat  \|\|\|\|\|\|\| \|\|\|\|\|\| \|\|\|\|\| \|\|\|\|\|\|\|\|\|\|\| tttttct-tgccat--taaaatcactcctaaat |
| 10F | 37831 | *Pseudomonas* phage Ab31 | *Pseudomonas* sp | cgcatgaagccggagaagtttgagcagttctg  \|\|\|\|\|\|\|\|\|\|\|\|\|\|\|\|\|\|\|\| \|\| \|\|\|\|\|\|\|\| cgcatgaagccggagaagttcgaacagttctg |
| 10F | 37243 | *Staphylococcus* phage StB20 | *Thermoanaerobacter* sp | tttttctctgccatattaaaa-cactcctaaat  \|\|\|\|\|\|\| \|\|\|\|\|\| \|\|\|\|\| \|\|\|\|\|\|\|\|\|\|\| tttttct-tgccat--taaaatcactcctaaat |
| 10F | 6955 | *Staphylococcus* phage CNPH82 | *Clostridioides difficile*  *Clostridium difficile*  *Thermodesulfobium narugense*  *Dictyoglomus turgidum* | gatgcatgtcatattcctaaacg  \|\|\|\|\| \|\|\|\|\|\|\|\|\|\|\|\|\|\|\|\|\| gatgc-tgtcatattcctaaacg  gatgcatgtcatattcctaaacg  \|\|\|\|\| \|\|\|\|\|\|\|\|\|\|\|\|\|\|\|\|\| gatgc-tgtcatattcctaaacg  aggaggacaaaaatgactaa  \|\|\|\|\|\|\|\|\|\|\|\|\|\|\|\|\|\|\|\| aggaggacaaaaatgactaa  aaaattaaa-agaggaagaagaagaaaaa  \|\|\|\|\|\|\|\|\| \|\|\| \|\|\|\|\|\|\|\|\|\|\|\|\|\| aaaattaaacaga--aagaagaagaaaaa |
| 10F | 6263 | *Salmonella* phage SEN34 | *Chlorobium limicola* | aatctggtctttcagcagcttgtg  \|\|\|\|\|\|\|\|\|\|\|\| \|\|\|\|\|\|\|\|\|\|\| aatctggtctttaagcagcttgtg |
| 10F | 6094 | *Pseudomonas* phage phiPSA1 | *Chloroflexus aurantiacus* | atacgaaaaagccccggcagataccg  \|\|\|\| \|\|\|\|\|\|\|\|\|\|\|\|\| \|\|\|\|\|\|\| atac-aaaaagccccggc-gataccg |
| 10S | 55049 | *Clostridium* phage phiMMP02 | *Clostridium botulinum*  *Clostridium perfringens*  *Fusobacterium hwasookii*  *Caldicellulosiruptor obsidiansis*  *Sebaldella termitidis* | ggttgtaaatgcctaatttattcccagaggatac  \|\|\|\|\|\|\|\|\|\| \|\|\|\|\|\|\|\|\|\|\|\|\|\|\|\|\| \|\|\|\|\| ggttgtaaatacctaatttattcccagaagatac  agaaggatttgagatag-tactaaag  \|\|\|\|\|\|\|\|\|\|\|\|\|\|\|\|\| \|\|\|\|\|\|\|\| agaaggatttgagatagctactaaag  agttagagaaa-aa-aca-aaatgattttaa  \|\|\|\|\|\|\|\|\|\|\| \|\| \|\|\| \|\|\|\|\|\|\|\|\|\|\|\| agttagagaaagaatacagaaatgattttaa  gagcttaaaagaaaagaaaaaaaga  \|\|\|\|\|\|\|\|\|\|\|\|\|\|\| \|\|\|\|\|\|\|\|\| gagcttaaaagaaaa-aaaaaaaga  attta-attaataagatagttaatattaatac  \|\|\|\|\| \|\|\| \|\|\|\|\|\|\|\|\|\| \|\|\|\|\|\|\|\|\|\| atttacattc-taagatagtt-atattaatac |
| 10S | 44057 | *Staphylococcus* phage phiNM3 | *Leptotrichia buccalis*  *Nitrosomonas ureae*  *Methanosaeta concilii* | atgattaaattatttatcatatctgaa  \|\|\|\|\| \|\|\|\|\|\|\|\|\| \|\|\|\|\|\|\|\|\|\|\| atgat-aaattattt-tcatatctgaa  cctg-ttatagaagggattaagac  \|\|\|\| \|\|\| \|\|\|\|\|\|\|\|\|\|\|\|\|\|\| cctgatta-agaagggattaagac  cccgatgaaaaggagaaattgaaa  \|\|\|\|\|\|\|\|\|\|\|\|\| \| \|\|\|\|\|\|\|\| cccgatgaaaagg-g-aattgaaa |
| 10S | 43445 | *Staphylococcus* phage phiRS7 | *Lactobacillus helveticus*  *Desulfurococcus kamchatkensis*  *Flavobacterium branchiophilum* | ttgttttaattcatctggaat  \|\|\|\|\|\|\|\|\|\|\|\|\|\|\|\|\|\|\|\|\| ttgttttaattcatctggaat  gtaaata-taatcaattgcttctttttctgtta  \|\|\|\|\|\|\| \| \|\|\|\|\|\|\|\|\|\|\|\|\|\|\|\|\| \|\|\|\| gtaaatact--tcaattgcttctttttc-gtta  tttaaaatttcta-tatt-tagat  \|\|\|\|\|\|\|\|\|\|\|\|\| \|\|\|\| \|\|\|\|\| tttaaaatttctactattgtagat |
| 10S | 40962 | *Streptococcus* phage EJ-1 | *Methanosarcina mazei*  *Desulfosporosinus orientis* | atac-acaagtacatcatcatcgtatt  \|\|\|\| \|\|\|\|\|\|\|\|\|\|\|\|\| \|\|\|\|\|\|\|\| atacgacaagtacatcat-atcgtatt  tggttgaaggaggaaaattga  \|\|\|\|\|\|\|\|\|\|\|\|\|\|\|\|\|\|\|\|\| tggttgaaggaggaaaattga |
| 10S | 39659 | *Staphylococcus* phage StB20 | *Thermosediminibacter oceani*  *Candidate division SR1 bacterium*  *Cyanothece sp* | gattgagtgtaaattaaatga  \|\|\|\|\|\|\|\|\|\|\|\|\|\|\|\|\|\|\|\|\| gattgagtgtaaattaaatga  atgatgaagcaga-tataaatttca-taaa  \|\|\|\| \|\|\|\| \|\|\| \|\|\|\|\|\|\|\|\|\|\| \|\|\|\| atga-gaag-agaatataaatttcattaaa  aaatcaattgaatggaaa  \|\|\|\|\|\|\|\|\|\|\|\|\|\|\|\|\|\| aaatcaattgaatggaaa |
| 10S | 17807 | *Bacillus* phage Fah | *Calothrix* sp  *Metallosphaera cuprina* | gttctccttgctcgccacgttctcctttttcacc  \|\|\|\|\|\|\|\|\| \|\|\| \|\|\|\|\| \|\|\|\|\|\|\|\|\|\|\|\|\|\| gttctcctttctctccacgctctcctttttcacc  tttctagttcttctattttattag  \|\|\|\|\|\|\|\|\|\|\|\| \|\|\|\|\|\|\|\|\|\|\| tttctagttctt-tattttattag |
| 10S | 17931 | *Pseudomonas* phage D3112 | *Clostridium perfringens* | ttatcattttcttatttttaaactctct  \|\|\|\|\|\|\| \|\|\|\|\| \|\|\|\|\|\|\|\|\|\|\|\|\|\| ttatcatattctt-tttttaaactctct |
| 10S | 14951 | *Streptococcus* phage PH15 | *Streptococcus salivarius*  *Streptococcus gordonii*  *Streptococcus* sp | gatttcagtttttggaagtcgtcttgacc  \|\|\|\|\| \|\|\|\|\|\|\|\|\|\|\|\|\|\|\|\|\|\|\|\|\|\|\| gatttaagtttttggaagtcgtcttgacc  gcgataaaatgcccgcgtagggcgtt  \|\|\|\|\|\|\|\|\|\|\|\|\|\| \|\|\|\|\|\|\|\|\|\|\| gcgataaaatgcccacgtagggcgtt  aactcaggagatacgtggagcttgcgtcct  \|\|\|\|\| \|\|\|\|\|\|\|\|\|\|\| \|\|\|\|\|\|\|\|\|\|\|\| aactctggagatacgtgtagcttgcgtcct |
| 10S | 13657 | *Staphylococcus* phage vB_SepiS-phiIPLA5 | *Staphylococcus schleiferi* | cgcccctctaaagataattcgattccctct  \|\|\|\|\|\|\|\|\|\|\|\|\|\|\|\|\|\|\|\|\|\|\|\|\|\|\|\|\|\| cgcccctctaaagataattcgattccctct |
| 10S | 7671 | *Vibrio* phage vB_VpaM_MAR | *Methanosarcina barkeri* | tcctatatttc-ttaaaacgttt  \|\|\|\|\|\|\|\|\|\|\| \|\|\|\|\|\|\|\|\|\|\| tcctatatttctttaaaacgttt |
| 10S | 6713 | *Staphylococcus* phage vB_SepiS-phiIPLA5 | *Methanocaldococcus bathoardescens* | cttttgttatataaatgaatgttt  \|\|\|\|\|\|\|\|\|\|\|\| \|\|\|\|\|\|\|\|\|\|\| cttttgttatat-aatgaatgttt |
| 10S | 5309 | *Staphylococcus* phage Stb20 | *Caldicellulosiruptor obsidiansis* | caagaacaattagataatttagaag-atta  \|\|\|\|\|\|\| \|\|\|\|\|\| \|\|\|\|\|\|\|\|\|\| \|\|\|\| caagaactattaga-aatttagaagaatta |
| 9F | 53525 | *Pseudomonas* phage phiPSA1 | *Pseudomonas parafulva*  *Pseudomonas parafulva*  *Pseudomonas parafulva* | tatccgccagcgcagagccgagcgcgcc  \|\|\|\|\|\|\|\|\|\| \|\|\|\|\|\|\|\|\|\|\|\|\|\|\|\|\| tatccgccagagcagagccgagcgcgcc  aatcaaccgccctggagggcaga  \|\|\|\|\|\|\|\|\|\|\|\|\|\|\|\|\|\|\|\|\|\|\| aatcaaccgccctggagggcaga  agctgcaggccggcaaccagcgccagctctc  \|\|\|\|\|\|\|\|\|\|\|\|\|\|\| \| \|\|\|\|\|\|\|\|\|\|\|\|\| agctgcaggccggcagcgagcgccagctctc |
| 9F | 41981 | *Staphylococcus* phage StB20 | *Streptococcus macedonicus*  *Thermoanaerobacter sp* | aatatacgaaaattcgtattag  \|\|\|\|\|\|\|\|\|\|\|\|\|\|\|\|\|\|\|\|\|\| aatatacgaaaattcgtattag  tttttctctgccatattaaaa-cactcctaaat  \|\|\|\|\|\|\| \|\|\|\|\|\| \|\|\|\|\| \|\|\|\|\|\|\|\|\|\|\| tttttct-tgccat--taaaatcactcctaaat |
| 9F | 36160 | *Pseudomonas* phage JBD25 | *Thermococcus eurythermalis* | gccgaggcgcttg-gggtgctgtc  \|\|\|\|\|\|\|\|\|\|\|\|\| \|\|\|\|\|\|\|\|\|\| gccgaggcgcttgagggtgctgtc |
| 9F | 33961 | *Synechococcus* phage S-CBS3 | *Actinomyces hongkongensis*  *Sorangium cellulosum* | gccgc-gattgcgacggctgccacgcc  \|\|\|\|\| \|\|\|\|\|\|\|\|\|\|\|\|\|\|\|\| \|\|\|\| gccgcagattgcgacggctgcc-cgcc  gccgccgcctttca-cggcgg  \|\|\|\|\|\|\|\|\|\|\|\|\|\| \|\|\|\|\|\| gccgccgcctttcagcggcgg |
| 9AX | 43032 | *Bacillus* phage Fah | *Methanosarcina mazei* | att-acagcagatcatatcaaagggcaagtttt  \|\|\| \|\|\|\| \| \|\|\|\|\|\|\|\|\|\|\|\|\|\|\|\|\| \|\|\|\| attgacag-a-atcatatcaaagggcaa-tttt |
| 9AX | 38103 | *Pseudomonas* phage Ab31 | *Pseudomonas* sp | cgcatgaagccggagaagtttgagcagttctg  \|\|\|\|\|\|\|\|\|\|\|\|\|\|\|\|\|\|\|\| \|\| \|\|\|\|\|\|\|\| cgcatgaagccggagaagttcgaacagttctg |
| 9AX | 22668 | *Pseudomonas* phage phiPSA1 | *Clostridium stercorarium* | gccctggccgccgccga-a-aagcaga  \|\|\|\|\|\|\|\|\|\|\|\|\|\|\|\|\| \| \|\|\|\|\|\|\| gccctggccgccgccgacagaagcaga |
| 9AX | 7508 | *Pseudomonas* phage phiPSA1 | *Pseudomonas chlororaphis*  *Pseudomonas chlororaphis* | tcccgctggttgccgatgggcgcgggg-gag  \|\|\|\|\|\|\|\|\|\|\|\|\|\|\|\|\|\|\|\|\|\|\|\|\|\|\| \|\|\| tcccgctggttgccgatgggcgcggggtgag  aggctgaggtggttgctgatcatgctgcc  \|\|\|\|\|\|\|\|\|\|\| \|\|\|\|\|\|\|\|\|\|\|\| \|\|\|\| aggctgaggtgattgctgatcatggtgcc |
| 8S | 53520 | *Pseudomonas* phage phiPSA1 | *Pseudomonas parafulva*  *Pseudomonas parafulva*  *Pseudomonas parafulva* | ggcgcgctcggctctgcgctggcggata  \|\|\|\|\|\|\|\|\|\|\|\|\|\|\|\|\| \|\|\|\|\|\|\|\|\|\| ggcgcgctcggctctgctctggcggata  tctgccctccagggcggttgatt  \|\|\|\|\|\|\|\|\|\|\|\|\|\|\|\|\|\|\|\|\|\|\| tctgccctccagggcggttgatt  gagagctggcgctggttgccggcctgcagct  \|\|\|\|\|\|\|\|\|\|\|\|\| \| \|\|\|\|\|\|\|\|\|\|\|\|\|\|\| gagagctggcgctcgctgccggcctgcagct |
| 8S | 37831 | *Pseudomonas* phage Ab31 | *Pseudomonas* sp | cagaactgctcaaacttctccggcttcatgcg  \|\|\|\|\|\|\|\| \|\| \|\|\|\|\|\|\|\|\|\|\|\|\|\|\|\|\|\|\|\| cagaactgttcgaacttctccggcttcatgcg |
| 8S | 24791 | *Pseudomonas* phage JBD25 | *Thermococcus eurythermalis* | gccgaggcgcttg-gggtgctgtc  \|\|\|\|\|\|\|\|\|\|\|\|\| \|\|\|\|\|\|\|\|\|\| gccgaggcgcttgagggtgctgtc |
| 8S | 10298 | *Pseudomonas* phage JBD25 | *Caldilinea aerophila* | gaaccggcgcaaccagcagctcagca  \|\|\|\|\|\| \| \|\|\|\|\|\|\|\|\|\|\|\|\|\|\|\|\| gaaccg-c-caaccagcagctcagca |
| 8S | 10286 | *Pseudomonas* phage phiPSA1 | *Clostridium stercorarium* | tctgctt-t-tcggcggcggccagggc  \|\|\|\|\|\|\| \| \|\|\|\|\|\|\|\|\|\|\|\|\|\|\|\|\| tctgcttctgtcggcggcggccagggc |
| 8F | 41536 | *Staphylococcus* phage StB20 | *Thermoanaerobacter* sp | atttaggagtg-ttttaatatggcagagaaaaa  \|\|\|\|\|\|\|\|\|\|\| \|\|\|\|\| \|\|\|\|\|\| \|\|\|\|\|\|\| atttaggagtgatttta--atggca-agaaaaa |
| 8F | 25915 | *Pseudomonas* phage JBD25 | *Thermococcus eurythermalis* | gccgaggcgcttg-gggtgctgtc  \|\|\|\|\|\|\|\|\|\|\|\|\| \|\|\|\|\|\|\|\|\|\| gccgaggcgcttgagggtgctgtc |
| 8F | 14482 | *Pseudomonas* phage Ab31 | *Pseudomonas* sp | cgcatgaagccggagaagtttgagcagttctg  \|\|\|\|\|\|\|\|\|\|\|\|\|\|\|\|\|\|\|\| \|\| \|\|\|\|\|\|\|\| cgcatgaagccggagaagttcgaacagttctg |
| 8F | 10357 | *Pseudomonas* phage JBD25 | *Caldilinea aerophila* | tgctgagctgctggttgcgccggttc  \|\|\|\|\|\|\|\|\|\|\|\|\|\|\|\|\| \| \|\|\|\|\|\| tgctgagctgctggttg-g-cggttc |
| 8AX | 48904 | *Pseudomonas* phage phiPSA1 | *Pseudomonas chlororaphis*  *Clostridium stercorarium*  *Pseudomonas chlororaphis* | tcccgctggttgccgatgggcgcgggg-gag  \|\|\|\|\|\|\|\|\|\|\|\|\|\|\|\|\|\|\|\|\|\|\|\|\|\|\| \|\|\| tcccgctggttgccgatgggcgcggggtgag  gccctggccgccgccga-a-aagcaga  \|\|\|\|\|\|\|\|\|\|\|\|\|\|\|\|\| \| \|\|\|\|\|\|\| gccctggccgccgccgacagaagcaga  aggctgaggtggttgctgatcatgctgcc  \|\|\|\|\|\|\|\|\|\|\| \|\|\|\|\|\|\|\|\|\|\|\| \|\|\|\| aggctgaggtgattgctgatcatggtgcc |
| 8AX | 41764 | *Staphylococcus* phage StB20 | *Streptococcus gallolyticus*  *Thermoanaerobacter* sp | tttatcccatactctaaattt  \|\|\|\|\|\|\|\|\|\|\|\|\|\|\|\|\|\|\|\|\| tttatcccatactctaaattt  tttttctctgccatattaaaa-cactcctaaat  \|\|\|\|\|\|\| \|\|\|\|\|\| \|\|\|\|\| \|\|\|\|\|\|\|\|\|\|\| tttttct-tgccat--taaaatcactcctaaat |
| 8AX | 40770 | *Burkholderia* phage Bcep176 | *Acinetobacter venetianus* | atgcgcaaaatgcagaagaaaaaccg  \|\|\|\|\| \|\|\|\|\|\|\|\|\|\|\|\|\|\|\|\|\|\|\|\| atgcgaaaaatgcagaagaaaaaccg |
| 8AX | 37831 | *Pseudomonas* phage Ab31 | *Pseudomonas* sp | cagaactgctcaaacttctccggcttcatgcg  \|\|\|\|\|\|\|\| \|\| \|\|\|\|\|\|\|\|\|\|\|\|\|\|\|\|\|\|\|\| cagaactgttcgaacttctccggcttcatgcg |
| 8AX | 26134 | *Pseudomonas* phage JBD25 | *Thermococcus eurythermalis* | gacagcaccc-caagcgcctcggc  \|\|\|\|\|\|\|\|\|\| \|\|\|\|\|\|\|\|\|\|\|\|\| gacagcaccctcaagcgcctcggc |
| 8AX | 16869 | *Thermus* phage phiOH2 | *Clostridium botulinum*  *Pleurocapsa* sp | tttaat-tt-ttaaatattttttctgt  \|\|\|\|\|\| \|\| \|\|\|\|\|\|\|\|\|\|\|\|\|\|\|\|\| tttaatcttattaaatattttttctgt  tccatat-aactaatttctctaat  \|\|\|\|\| \| \|\|\|\|\|\|\|\|\|\|\|\|\|\|\|\| tccat-tcaactaatttctctaat |
| 8AX | 7394 | *Salmonella* phage SEN34 | *Chlorobium limicola* | aatctggtctttcagcagcttgtg  \|\|\|\|\|\|\|\|\|\|\|\| \|\|\|\|\|\|\|\|\|\|\| aatctggtctttaagcagcttgtg |
| 7S | 49229 | *Pseudomonas* phage phiPSA1 | *Pseudomonas chlororaphis*  *Clostridium stercorarium*  *Pseudomonas chlororaphis* | ctc-ccccgcgcccatcggcaaccagcggga  \|\|\| \|\|\|\|\|\|\|\|\|\|\|\|\|\|\|\|\|\|\|\|\|\|\|\|\|\|\| ctcaccccgcgcccatcggcaaccagcggga  tctgctt-t-tcggcggcggccagggc  \|\|\|\|\|\|\| \| \|\|\|\|\|\|\|\|\|\|\|\|\|\|\|\|\| tctgcttctgtcggcggcggccagggc  ggcagcatgatcagcaaccacctcagcct  \|\|\|\| \|\|\|\|\|\|\|\|\|\|\|\| \|\|\|\|\|\|\|\|\|\|\| ggcaccatgatcagcaatcacctcagcct |
| 7S | 41944 | Staphylococcus phage StB20 | *Streptococcus gallolyticus*  *Thermoanaerobacter sp* | aaatttagagtatgggataaa  \|\|\|\|\|\|\|\|\|\|\|\|\|\|\|\|\|\|\|\|\| aaatttagagtatgggataaa  atttaggagtg-ttttaatatggcagagaaaaa  \|\|\|\|\|\|\|\|\|\|\| \|\|\|\|\| \|\|\|\|\|\| \|\|\|\|\|\|\| atttaggagtgatttta--atggca-agaaaaa |
| 7S | 37831 | *Pseudomonas* phage Ab31 | *Pseudomonas* sp | cgcatgaagccggagaagtttgagcagttctg  \|\|\|\|\|\|\|\|\|\|\|\|\|\|\|\|\|\|\|\| \|\| \|\|\|\|\|\|\|\| cgcatgaagccggagaagttcgaacagttctg |
| 7S | 36350 | *Pseudomonas* phage JBD25 | *Thermococcus eurythermalis* | gccgaggcgcttg-gggtgctgtc  \|\|\|\|\|\|\|\|\|\|\|\|\| \|\|\|\|\|\|\|\|\|\| gccgaggcgcttgagggtgctgtc |
| 7S | 26959 | *Bacillus* phage phBC6A52 | *Geobacillus thermoleovorans* | catcgtattcagaagggaatctct-tagctctacaa  \|\|\|\|\|\|\|\|\|\| \|\|\|\|\|\|\|\| \|\| \| \|\|\|\|\|\|\|\|\|\|\| catcgtattccgaagggaacct-tctagctctacaa |
| 7S | 20117 | *Staphylococcus* phage 6ec | *Thermodesulfobium narugense* | tgatttcatctttgatattaa  \|\|\|\|\|\|\|\|\|\|\|\|\|\|\|\|\|\|\|\|\| tgatttcatctttgatattaa |
| 7S | 17786 | *Staphylococcus* phage phiETA3 | *Staphylococcus aureus*  *Staphylococcus aureus* | gcccatttaattaattcatctaatctcatttctt  \|\|\|\|\| \|\|\|\|\|\|\|\|\|\|\|\|\|\|\|\| \|\|\|\|\|\|\|\|\|\|\| gcccacttaattaattcatctagtctcatttctt  taaacctttgattgctcttagttttagttatgt  \|\|\|\| \|\|\|\|\|\|\|\|\|\|\|\|\|\|\|\| \| \|\|\|\|\|\|\|\|\| taaatctttgattgctcttagctctagttatgt |
| 7S | 16872 | *Staphylococcus* phage phiRS7 | *Thermoanaerobacterium thermosaccharolyticum* | ttaatgcaatacacaggactt-aaagataagaacgg  \|\|\|\|\|\|\|\|\|\|\|\|\|\|\|\|\| \|\|\| \|\|\|\|\|\|\|\| \|\|\|\|\| ttaatgcaatacacagg-cttaaaagataaaaacgg |
| 7S | 16752 | *Staphylococcus* phage phiETA3 | *Staphylococcus agnetis* | ggtgccttttgttctgcaattgaagttat  \|\|\|\|\|\|\|\|\|\|\|\| \|\|\|\|\|\|\|\|\|\|\|\|\|\|\| ggtgccttttgtagtgcaattgaagttat |
| 7S | 13110 | *Mycobacterium* phage Che9d | *Haliangium ochraceum* | ggacccgg-acccaccggacccg  \|\|\|\|\|\|\|\| \|\|\|\|\|\|\|\|\|\|\|\|\|\| ggacccgggacccaccggacccg |
| 7S | 7278 | *Mycobacterium* phage DNAIII | *Natrinema* sp | atcgcctcgcgacgccacgcagcc  \|\|\|\|\| \|\|\|\|\|\|\|\|\|\|\|\|\|\|\|\|\|\| atcgc-tcgcgacgccacgcagcc |
| 7F | 49331 | *Pseudomonas* phage phiPSA1 | *Pseudomonas chlororaphis*  *Clostridium stercorarium*  *Pseudomonas chlororaphis* | tcccgctggttgccgatgggcgcgggg-gag  \|\|\|\|\|\|\|\|\|\|\|\|\|\|\|\|\|\|\|\|\|\|\|\|\|\|\| \|\|\| tcccgctggttgccgatgggcgcggggtgag  gccctggccgccgccga-a-aagcaga  \|\|\|\|\|\|\|\|\|\|\|\|\|\|\|\|\| \| \|\|\|\|\|\|\| gccctggccgccgccgacagaagcaga  aggctgaggtggttgctgatcatgctgcc  \|\|\|\|\|\|\|\|\|\|\| \|\|\|\|\|\|\|\|\|\|\|\| \|\|\|\| aggctgaggtgattgctgatcatggtgcc |
| 7F | 48462 | *Bacillus* phage phBC6A52 | *Sulfolobus islandicus*  *Thermoanaerobacterium xylanolyticum* | aaaataagggataaa-acatagga  \|\|\|\|\|\|\|\|\|\|\|\|\|\|\| \|\|\|\|\|\|\|\| aaaataagggataaacacatagga  tattt-tttatttgtc-agtaattgtc  \|\|\|\|\| \|\|\|\|\|\|\|\|\|\| \|\|\|\|\|\|\|\|\|\| tatttctttatttgtcaagtaattgtc |
| 7F | 43467 | *Staphylococcus* phage phiRS7 | *Thermodesulfobium narugense*  *Fervidobacterium nodosum* | tgatttcatctttgatattaa  \|\|\|\|\|\|\|\|\|\|\|\|\|\|\|\|\|\|\|\|\| tgatttcatctttgatattaa  tctttgtcttttcttttttatttaacaaa--caaaa  \|\|\|\|\| \|\|\|\|\|\|\|\|\|\|\|\| \|\|\|\|\|\|\|\|\| \|\|\|\|\| tcttt-tcttttcttttt--tttaacaaaagcaaaa |
| 7F | 40754 | *Burkholderia* phage Bcep176 | *Acinetobacter venetianus* | cggtttttcttctgcattttgcgcat  \|\|\|\|\|\|\|\|\|\|\|\|\|\|\|\|\|\|\|\| \|\|\|\|\| cggtttttcttctgcatttttcgcat |
| 7F | 40473 | *Staphylococcus* phage2638A | *Staphylococcus schleiferi*  *Staphylococcus aureus*  *Staphylococcus schleiferi* | atatactcccttgccatgtattaattctggaccac  \|\|\|\|\|\|\|\|\| \|\| \|\|\|\|\|\|\|\|\|\|\|\|\|\|\|\|\|\|\|\|\|\| atatactcctttaccatgtattaattctggaccac  atatactcccttgccatgtattaattctggaccac  \|\|\|\|\|\|\|\|\| \|\| \|\|\|\|\|\|\|\|\|\|\|\|\|\|\|\|\|\|\|\|\|\| atatactcctttaccatgtattaattctggaccac  atttcgttaaatttattaatgtaagccatt  \|\|\|\|\|\|\|\| \|\|\|\|\| \|\|\|\|\|\|\|\|\|\|\|\|\|\|\| atttcgttgaatttgttaatgtaagccatt |
| 7F | 37831 | *Pseudomonas* phage Ab31 | *Pseudomonas* sp | cagaactgctcaaacttctccggcttcatgcg  \|\|\|\|\|\|\|\| \|\| \|\|\|\|\|\|\|\|\|\|\|\|\|\|\|\|\|\|\|\| cagaactgttcgaacttctccggcttcatgcg |
| 7F | 37302 | *Pseudomonas* phage JBD25 | *Thermococcus eurythermalis* | gccgaggcgcttg-gggtgctgtc  \|\|\|\|\|\|\|\|\|\|\|\|\| \|\|\|\|\|\|\|\|\|\| gccgaggcgcttgagggtgctgtc |
| 7F | 21581 | *Pseudomonas* phage phiPSA1 | *Pseudomonas parafulva*  *Pseudomonas parafulva*  *Pseudomonas chlororaphis* | ggcgcgctcggctctgcgctggcggata  \|\|\|\|\|\|\|\|\|\|\|\|\|\|\|\|\| \|\|\|\|\|\|\|\|\|\| ggcgcgctcggctctgctctggcggata  gagagctggcgctggttgccggcctgcagct  \|\|\|\|\|\|\|\|\|\|\|\|\| \| \|\|\|\|\|\|\|\|\|\|\|\|\|\|\| gagagctggcgctcgctgccggcctgcagct  agcgcatcccgctcggccagctggg  \|\|\|\|\|\|\|\|\|\|\|\|\|\|\|\|\|\|\| \|\|\|\|\| agcgcatcccgctcggccacctggg |
| 7F | 21262 | *Pseudomonas* phage phiPSA1 | *Pseudomonas parafulva* | aatcaaccgccctggagggcaga  \|\|\|\|\|\|\|\|\|\|\|\|\|\|\|\|\|\|\|\|\|\|\| aatcaaccgccctggagggcaga |
| 7F | 20227 | *Staphylococcus* phage StB20 | *Methanosarcina lacustris*  *Thermoanaerobacter* sp | attcataatttctttgttatctataatcgc  \|\|\| \|\|\| \|\|\| \|\|\|\|\|\|\|\|\|\|\|\|\|\|\|\|\|\| att-ata-ttt-tttgttatctataatcgc  tttttctctgccatattaaaa-cactcctaaat  \|\|\|\|\|\|\| \|\|\|\|\|\| \|\|\|\|\| \|\|\|\|\|\|\|\|\|\|\| tttttct-tgccat--taaaatcactcctaaat |
| 7F | 17478 | *Staphylococcus* phage 2368A | *Methanosarcina mazei* | tttttttatgttcaaaaaaataat  \|\|\|\|\|\|\|\|\|\|\| \|\|\|\|\|\|\|\|\|\|\|\| tttttttatgt-caaaaaaataat |
| 7F | 15604 | *Lactobacillus* phage A2 | *Staphylococcus schleiferi*  *Staphylococcus aureus*  *Lactobacillus helveticus* | atatactcctttgccatag-attaattctggaccac  \|\|\|\|\|\|\|\|\|\|\|\| \|\|\|\| \| \|\|\|\|\|\|\|\|\|\|\|\|\|\|\|\| atatactcctttaccat-gtattaattctggaccac  atatactcctttgccatag-attaattctggaccac  \|\|\|\|\|\|\|\|\|\|\|\| \|\|\|\| \| \|\|\|\|\|\|\|\|\|\|\|\|\|\|\|\| atatactcctttaccat-gtattaattctggaccac  ttgttttaattcatctggaat  \|\|\|\|\|\|\|\|\|\|\|\|\|\|\|\|\|\|\|\|\| ttgttttaattcatctggaat |
| 7F | 9665 | *Lactobacillus* phage A2 | *Lactobacillus helveticus*  *Treponema pedis* | attccagatgaattaaaacaa  \|\|\|\|\|\|\|\|\|\|\|\|\|\|\|\|\|\|\|\|\| attccagatgaattaaaacaa  taatattccagatgaattaa  \|\|\|\|\|\|\|\|\|\|\|\|\|\|\|\|\|\|\|\| taatattccagatgaattaa |
| 7F | 8093 | *Salmonella* phage SEN34 | *Syntrophobacter fumaroxidans* | accatgagtaaatacaccgacc  \|\|\|\|\|\|\|\|\|\|\|\|\|\|\|\|\|\|\|\|\|\| accatgagtaaatacaccgacc |
| 7F | 4912 | *Salmonella* phage SEN34 | *Chlorobium limicola* | aatctggtctttcagcagcttgtg  \|\|\|\|\|\|\|\|\|\|\|\| \|\|\|\|\|\|\|\|\|\|\| aatctggtctttaagcagcttgtg |
| 7F | 6750 | *Staphylococcus* phage StB20 | *Moorella thermoacetica* | ctaaatattcttcaggtgt-tacaaatcctcttt  \|\|\|\|\|\|\|\|\|\|\|\|\|\|\|\| \|\| \|\| \|\|\|\|\|\|\| \|\|\| ctaaatattcttcaggagtgta-aaatcct-ttt |
| 7AX | 141510 | *Staphylococcus* phage phiIPLA-C1C | *Moraxella ovis*  *Fervidobacterium pennivorans*  *Thermofilum sp*  *Desulfobacter postgatei*  *Clostridium saccharoperbutylacetonicum*  *Mesotoga prima*  *Saprospira grandis*  *Bacteroides* sp | atttttttatttattttttt-ttatatttt  \|\|\|\|\|\|\|\|\|\|\|\|\|\|\|\|\|\|\|\| \|\| \|\|\|\|\|\| atttttttatttatttttttatt-tatttt  acc-cctttttttaatttttttattttt  \|\|\| \|\|\|\|\|\|\|\|\|\| \|\|\|\|\|\|\|\|\|\|\|\|\| acctccttttttta-tttttttattttt  atttttattg-atttacttatttctttttt-atct  \|\|\|\|\|\|\|\|\|\| \|\|\|\| \|\|\|\|\|\|\|\|\|\|\|\|\| \|\|\|\| atttttattgcatttt-ttatttcttttttcatct  taatttaaaaaataaaggagtaaaaagtaatg  \|\|\|\|\|\|\|\| \|\|\|\|\|\|\|\|\|\|\| \|\|\|\|\| \|\|\|\|\| taatttaacaaataaaggag-aaaaa-taatg  tataaattctt-caattcttttaatttcttc  \|\|\|\|\| \|\|\|\|\| \|\|\|\|\|\|\|\|\| \|\|\|\|\|\|\|\|\| tataa-ttctttcaattcttt-aatttcttc  ataaaaaaggagtgtaaaaatatgaata  \|\|\|\|\|\|\|\|\|\|\|\| \| \|\|\|\|\|\|\|\|\|\|\|\|\| ataaaaaaggag-g-aaaaatatgaata  cttcaattcttttaatttcttcttt  \|\|\|\|\|\|\|\|\|\|\|\|\|\|\|\|\|\|\|\|\| \|\|\| cttcaattcttttaatttctt-ttt  tttg-tttcataaattttatctttgaca  \|\|\|\| \|\|\|\|\| \|\|\|\|\|\|\|\|\|\|\|\|\|\|\|\|\| tttgctttca-aaattttatctttgaca |
| 7AX | 49489 | *Pseudomonas* phage phiPSA1 | *Pseudomonas chlororaphis*  *Clostridium stercorarium*  *Pseudomonas chlororaphis* | ctc-ccccgcgcccatcggcaaccagcggga  \|\|\| \|\|\|\|\|\|\|\|\|\|\|\|\|\|\|\|\|\|\|\|\|\|\|\|\|\|\| ctcaccccgcgcccatcggcaaccagcggga  tctgctt-t-tcggcggcggccagggc  \|\|\|\|\|\|\| \| \|\|\|\|\|\|\|\|\|\|\|\|\|\|\|\|\| tctgcttctgtcggcggcggccagggc  ggcagcatgatcagcaaccacctcagcct  \|\|\|\| \|\|\|\|\|\|\|\|\|\|\|\| \|\|\|\|\|\|\|\|\|\|\| ggcaccatgatcagcaatcacctcagcct |
| 7AX | 44202 | *Salmonella* phage SEN34 | *Escherichia coli*  *Syntrophobacter fumaroxidans* | tcaccaaatcagtgacaaaaagtgtcaccaaa  \|\|\|\|\|\|\|\| \|\|\|\|\|\|\|\|\|\|\|\| \|\|\|\|\|\|\|\|\|\| tcaccaaaacagtgacaaaaactgtcaccaaa  accatgagtaaatacaccgacc  \|\|\|\|\|\|\|\|\|\|\|\|\|\|\|\|\|\|\|\|\|\| accatgagtaaatacaccgacc |
| 7AX | 40798 | *Burkholderia* phage Bcep176 | *Acinetobacter venetianus* | cggtttttcttctgcattttgcgcat  \|\|\|\|\|\|\|\|\|\|\|\|\|\|\|\|\|\|\|\| \|\|\|\|\| cggtttttcttctgcatttttcgcat |
| 7AX | 37833 | *Pseudomonas* phage Ab31 | *Pseudomonas* sp | cgcatgaagccggagaagtttgagcagttctg  \|\|\|\|\|\|\|\|\|\|\|\|\|\|\|\|\|\|\|\| \|\| \|\|\|\|\|\|\|\| cgcatgaagccggagaagttcgaacagttctg |
| 7AX | 36416 | *Pseudomonas* phage JBD25 | *Thermococcus eurythermalis* | gccgaggcgcttg-gggtgctgtc  \|\|\|\|\|\|\|\|\|\|\|\|\| \|\|\|\|\|\|\|\|\|\| gccgaggcgcttgagggtgctgtc |
| 3S | 31602 | *Pseudomonas* phage phiCTX | *Pseudomonas balearica*  *Pseudomonas balearica*  *Pseudomonas aeruginosa*  *Pseudomonas pseudoalcaligenes*  *Pseudomonas balearica*  *Pseudomonas stutzeri*  *Pseudomonas balearica*  *Pseudomonas balearica*  *Pseudomonas* sp  *Marichromatium purpuratum*  *Marichromatium purpuratum*  *Pseudomonas stutzeri*  *Burkholderia multivorans*  *Burkholderia multivorans*  *Burkholderia multivorans* | ccactccggcagcccgtacacctcctggttga  \|\|\|\|\|\|\|\|\|\|\|\|\|\|\|\|\|\|\|\|\|\|\|\|\|\|\|\|\|\|\|\| ccactccggcagcccgtacacctcctggttga  caactgtcgcggaacgcctcctcgagcagctc  \|\|\|\|\|\|\|\|\|\|\|\|\|\| \|\|\|\|\|\|\|\|\|\|\|\|\|\|\|\|\| caactgtcgcggaaggcctcctcgagcagctc  ttcgccttcgagaactacacccgcaccgccca  \|\|\|\|\| \|\|\|\|\|\|\|\|\|\|\|\|\|\|\|\|\|\|\|\|\|\|\|\|\|\| ttcgcattcgagaactacacccgcaccgccca  tacgaagccatcctcgccgagcgcaaggcc  \|\|\|\|\|\| \|\|\|\|\|\|\|\|\|\|\|\|\|\|\|\|\|\|\|\|\|\|\| tacgaaaccatcctcgccgagcgcaaggcc  ggcctgcgccggtggatggggcaggt  \|\|\|\|\|\|\|\|\|\|\|\|\|\|\|\|\|\|\|\|\|\|\|\|\|\| ggcctgcgccggtggatggggcaggt  tgggcggtggacaagcccatgcacgcc-tccctggt  \|\|\|\|\| \|\|\|\|\|\|\|\|\|\|\|\|\|\|\|\|\|\| \|\| \|\|\|\|\|\|\|\| tgggcagtggacaagcccatgcac-ccgtccctggt  gccagtcggtgccgaactcttcggcactgatc  \|\|\|\|\|\| \|\| \|\|\|\|\|\|\|\|\|\|\|\|\|\|\|\|\|\|\|\|\|\| gccagttggcgccgaactcttcggcactgatc  ctgttcggccgcccggacgaagagggcaagga  \|\|\|\|\| \|\|\|\|\|\|\|\|\|\|\|\|\|\| \|\|\|\|\|\|\|\|\|\|\| ctgtttggccgcccggacgaggagggcaagga  tcgtcgacgaactcgcacatca  \|\|\|\|\|\|\|\|\|\|\|\|\|\|\|\|\|\|\|\|\|\| tcgtcgacgaactcgcacatca  cacagcccc-gcgaatgcgccccgcgcg  \|\|\|\| \|\|\|\| \|\|\|\|\|\|\|\|\|\|\|\|\|\|\|\|\|\| caca-ccccagcgaatgcgccccgcgcg  aaggacgagcccgaatacgaccgcatcgcc  \|\|\|\|\|\|\|\|\|\|\|\|\|\| \|\|\|\|\|\|\|\| \|\|\|\|\|\| aaggacgagcccgagtacgaccgtatcgcc  atcgacaacggcagcc-tgcgcagcaccgccgcgcc  \|\|\|\|\|\|\|\|\|\|\|\|\| \|\| \| \|\|\|\|\|\|\|\|\|\|\| \|\|\|\|\| atcgacaacggca-ccatccgcagcaccgcggcgcc  cgccgagcgcgccgccga  \|\|\|\|\|\|\|\|\|\|\|\|\|\|\|\|\|\|  cgccgagcgcgccgccga  cgccgagcgcgccgccga  \|\|\|\|\|\|\|\|\|\|\|\|\|\|\|\|\|\|  cgccgagcgcgccgccga  ccgcgccgccgccgag-gc-ccgc  \|\|\|\|\|\|\|\|\|\|\|\|\|\|\|\| \|\| \|\|\|\|  ccgcgccgccgccgagcgcgccgc |
| 3S | 7213 | *Staphylococcus* phage StB20 | *Thermoanaerobacter* sp  *Methanosarcina lacustris* | tttttctctgccatattaaaa-cactcctaaat  \|\|\|\|\|\|\| \|\|\|\|\|\| \|\|\|\|\| \|\|\|\|\|\|\|\|\|\|\| tttttct-tgccat--taaaatcactcctaaat  atttctttgttatctataatcgc  \|\|\|\| \|\|\|\|\|\|\|\|\|\|\|\|\|\|\|\|\|\| attt-tttgttatctataatcgc |
| 3S | 53520 | *Pseudomonas* phage phiPSA1 | *Pseudomonas parafulva*  *Pseudomonas parafulva*  *Pseudomonas parafulva* | ggcgcgctcggctctgcgctggcggata  \|\|\|\|\|\|\|\|\|\|\|\|\|\|\|\|\| \|\|\|\|\|\|\|\|\|\| ggcgcgctcggctctgctctggcggata  tctgccctccagggcggttgatt  \|\|\|\|\|\|\|\|\|\|\|\|\|\|\|\|\|\|\|\|\|\|\| tctgccctccagggcggttgatt  gagagctggcgctggttgccggcctgcagct  \|\|\|\|\|\|\|\|\|\|\|\|\| \| \|\|\|\|\|\|\|\|\|\|\|\|\|\|\| gagagctggcgctcgctgccggcctgcagct |
| 3S | 36505 | *Pseudomonas* phage JBD25 | *Thermococcus eurythermalis* | gccgaggcgcttg-gggtgctgtc  \|\|\|\|\|\|\|\|\|\|\|\|\| \|\|\|\|\|\|\|\|\|\| gccgaggcgcttgagggtgctgtc |
| 3S | 44379 | *Salmonella* phage BP12C | *Acinetobacter baumannii* | atttcaggcaataaaaaaccc  \|\|\|\|\|\|\|\|\|\|\|\|\|\|\|\|\|\|\|\|\| atttcaggcaataaaaaaccc |
| 3S | 45342 | *Streptococcus* phage 20617 | *Lactobacillus helveticus*  *Streptococcus thermophilus*  *Streptococcus thermophilus*  *Streptococcus thermophilus*  *Streptococcus thermophilus*  *Streptococcus thermophilus*  *Streptococcus thermophilus*  *Streptococcus thermophilus*  *Streptococcus thermophilus*  *Streptococcus thermophiles*  *Myroides odoratus*  *Weeksella virosa* | actttaggcttagctgccatttgagtggcttg  \|\|\|\|\|\|\|\|\|\|\|\|\|\| \|\|\|\|\|\|\|\|\|\|\|\|\|\|\|\|\| actttaggcttagccgccatttgagtggcttg  aattcttcatccggtaactgctcaag  \|\|\|\|\|\|\|\|\|\|\|\|\|\|\|\|\|\|\|\|\|\|\|\|\|\| aattcttcatccggtaactgctcaag  cgatatttaaaatcattttcattacttca  \|\|\|\|\|\|\|\|\|\|\|\|\|\|\|\|\|\|\|\|\|\| \|\|\|\|\|\| cgatatttaaaatcattttcataacttca  gtttcttgttt-cttgtttgaccaaatcca  \|\|\|\|\|\| \|\|\|\| \|\|\|\|\|\|\|\|\|\|\|\|\|\|\|\|\|\| gtttct-gtttgcttgtttgaccaaatcca  aagttgtcatcaatcattactcc  \|\|\|\|\|\|\|\|\|\|\|\|\|\|\|\|\|\|\|\|\|\|\| aagttgtcatcaatcattactcc  caagtcgatttcccaaacttcgacct  \|\|\|\|\|\| \|\|\|\|\|\|\|\|\|\|\|\|\|\|\|\|\|\|\| caagtcaatttcccaaacttcgacct  ccaaagtttgattatagagcta  \|\|\|\|\|\|\|\|\|\|\|\|\|\|\|\|\|\|\|\|\|\| ccaaagtttgattatagagcta  accttgttttgaagtgcttggaagt  \|\|\|\|\|\|\|\|\|\|\|\|\|\| \|\|\|\|\|\|\|\|\|\| accttgttttgaagcgcttggaagt  tccaaaatcaactctttgatgtcgttatt  \|\|\|\|\|\|\|\| \|\| \|\|\|\|\|\|\|\|\|\|\|\|\|\|\|\|\| tccaaaattaattctttgatgtcgttatt  tgaatagtctttaaaccgcatttgaagcatata  \|\|\|\|\|\|\|\|\|\|\|\| \|\| \|\|\|\|\|\|\|\|\|\| \|\|\|\|\|\| tgaatagtctttgaatcgcatttgaaccatata  attcctttcaaattttagctagattgtag  \|\|\|\|\|\|\|\|\|\|\|\|\|\|\|\| \| \|\|\|\|\|\| \|\|\| attcctttcaaatttt-g-tagatt-tag  attcctttcaaattttagctagatt  \|\|\|\|\|\|\|\|\|\|\|\|\|\|\|\| \| \|\|\|\|\|\| attcctttcaaatttttg-tagatt |
| 3S | 37831 | *Pseudomonas* phage Ab31 | *Pseudomonas* sp | cagaactgctcaaacttctccggcttcatgcg  \|\|\|\|\|\|\|\| \|\| \|\|\|\|\|\|\|\|\|\|\|\|\|\|\|\|\|\|\|\| cagaactgttcgaacttctccggcttcatgcg |
| 3S | 12559 | *Vibrio* phage VP882 | *Pseudomonas balearica*  *Pseudomonas stutzeri*  *Pseudomonas pseudoalcaligenes* | gcggcgctcccagcgctcgagtgcgccgaggc  \|\|\|\|\|\|\|\|\|\|\|\|\|\|\|\|\|\|\|\|\| \|\|\|\|\|\|\|\|\|\| gcggcgctcccagcgctcgagggcgccgaggc  gtctcgctgacctcgccggccgctggg  \|\|\|\|\|\|\|\|\|\|\|\|\|\|\|\|\|\|\| \|\|\|\|\|\|\| gtctcgctgacctcgccgg-cgctggg  tccggtcgagccgttgcccgtcgatttcgtag  \|\|\|\|\|\|\|\|\|\|\|\|\| \|\| \|\|\|\|\|\|\|\| \|\|\|\|\|\| tccggtcgagccgctggccgtcgatctcgtag |
| 3S | 4037 | *Staphylococcus* phage StB20 | *Methanosarcina lacustris*  *Thermoanaerobacter* sp | gcgattatagataacaaagaaattatgaat  \|\|\|\|\|\|\|\|\|\|\|\|\|\|\|\|\|\| \|\|\| \|\|\| \|\|\| gcgattatagataacaaa-aaa-tat-aat  atttaggagtg-ttttaatatggcagagaaaaa  \|\|\|\|\|\|\|\|\|\|\| \|\|\|\|\| \|\|\|\|\|\| \|\|\|\|\|\|\| atttaggagtgatttta--atggca-agaaaaa |
| 3S | 7738 | *Staphylococcus* phage PH15 | *Methanosarcina mazei*  *Methanotorris igneus* | aagaagttg-aaaagaaaatttg  \|\|\|\|\|\|\|\|\| \|\|\|\|\|\|\|\|\|\|\|\|\| aagaagttggaaaagaaaatttg  ttgagtg-ttccgatgtgtatgctaaaaaa  \|\|\|\| \|\| \|\|\|\|\|\|\|\| \|\|\|\|\|\|\|\|\|\|\|\|\| ttga-tgattccgatgagtatgctaaaaaa |
| 3S | 34508 | *Pseudomonas* phage phiPSA1 | *Pseudomonas chlororaphis*  *Clostridium stercorarium*  *Pseudomonas chlororaphis* | tcccgctggttgccgatgggcgcgggg-gag  \|\|\|\|\|\|\|\|\|\|\|\|\|\|\|\|\|\|\|\|\|\|\|\|\|\|\| \|\|\| tcccgctggttgccgatgggcgcggggtgag  gccctggccgccgccga-a-aagcaga  \|\|\|\|\|\|\|\|\|\|\|\|\|\|\|\|\| \| \|\|\|\|\|\|\| gccctggccgccgccgacagaagcaga  aggctgaggtggttgctgatcatgctgcc  \|\|\|\|\|\|\|\|\|\|\| \|\|\|\|\|\|\|\|\|\|\|\| \|\|\|\| aggctgaggtgattgctgatcatggtgcc |
| 3S | 42828 | *Staphylococcus* phage IME_SA4 | *Methanosarcina mazei*  *Clostridium cellulolyticum* | aataaactacaaactgaa-ttgaa  \|\|\|\|\|\|\|\|\|\|\|\|\|\|\|\|\|\| \|\|\|\|\| aataaactacaaactgaatttgaa  gagcct-aattggagctataac  \|\|\|\|\|\| \|\|\|\|\|\|\|\|\|\|\|\|\|\|\| gagcctgaattggagctataac |
| 3S | 18598 | *Vibrio* phage VP882 | *Pseudomonas aeruginosa*  *Pseudomonas pseudoalcaligenes* | ttcccg-cagcacctcgaggcgctgcgcga  \|\|\|\|\|\| \| \|\|\|\|\|\|\|\|\|\|\|\|\|\|\|\|\|\|\|\|\| ttcccggc-gcacctcgaggcgctgcgcga  agtgggcgaatcgcctcgaggcgctgg  \|\|\|\|\|\|\|\|\|\| \|\|\|\|\|\|\|\|\|\|\|\|\|\|\|\| agtgggcgaaccgcctcgaggcgctgg |
| 3S | 13361 | *Streptococcus* phage phiARI0468_1 | *Streptococcus* sp  *Moraxella ovis* | tccagaccatgggtattatcataaagaccc  \|\|\|\|\|\|\|\|\|\|\|\| \|\| \|\|\|\|\|\|\|\|\|\|\|\|\|\| tccagaccatggatactatcataaagaccc  aaataa-taaaaaaataaataatataaatataa  \|\|\|\|\|\| \|\|\|\|\|\|\|\|\|\|\|\|\|\|\| \| \|\|\| \|\|\|\| aaataaataaaaaaataaataa-a-aaa-ataa |
| 3F | 70599 | *Paenibacillus* phage Davies | *Paenibacillus riograndensis* | cgggaacgctttgaatttagagtata  \|\|\|\|\|\|\|\| \|\|\|\|\|\|\|\|\|\|\|\|\|\|\|\|\| cgggaacggtttgaatttagagtata |
| 3F | 53712 | *Pseudomonas* phage phiPSA1 | *Pseudomonas parafulva*  *Pseudomonas parafulva*  *Pseudomonas parafulva* | tatccgccagcgcagagccgagcgcgcc  \|\|\|\|\|\|\|\|\|\| \|\|\|\|\|\|\|\|\|\|\|\|\|\|\|\|\| tatccgccagagcagagccgagcgcgcc  aatcaaccgccctggagggcaga  \|\|\|\|\|\|\|\|\|\|\|\|\|\|\|\|\|\|\|\|\|\|\| aatcaaccgccctggagggcaga  agctgcaggccggcaaccagcgccagctctc  \|\|\|\|\|\|\|\|\|\|\|\|\|\|\| \| \|\|\|\|\|\|\|\|\|\|\|\|\| agctgcaggccggcagcgagcgccagctctc |
| 3F | 35599 | *Bacillus* phage phBC6A52 | *Moraxella ovis* | tattatttttttatttatttatttt  \|\|\|\|\|\|\|\|\|\|\|\|\|\|\|\|\|\|\|\| \|\|\|\| tattatttttttatttattt-tttt |
| 3F | 12395 | *Pseudomonas* phage JBD25 | *Thermococcus eurythermalis* | gacagcaccc-caagcgcctcggc  \|\|\|\|\|\|\|\|\|\| \|\|\|\|\|\|\|\|\|\|\|\|\| gacagcaccctcaagcgcctcggc |
| 3F | 10196 | *Pseudomonas* phage JBD25 | *Caldilinea aerophila* | gaaccggcgcaaccagcagctcagca  \|\|\|\|\|\| \| \|\|\|\|\|\|\|\|\|\|\|\|\|\|\|\|\| gaaccg-c-caaccagcagctcagca |
| 2AX | 12810 | *Staphylococcus* phage 2368A | *Arthrospira platensis*  *Arthrospira platensis*  *Methanocaldococcus* sp  *Methanolinea tarda* | attt-tttatttatttattt-tttaatttg  \|\|\|\| \|\|\|\|\|\|\|\|\|\|\|\|\|\|\| \|\|\| \|\|\|\|\| atttatttatttatttatttattt-atttg  attt-tttatttatttattt-tttaattt  \|\|\|\| \|\|\|\|\|\|\|\|\|\|\|\|\|\|\| \|\|\| \|\|\|\| atttatttatttatttatttattt-attt  cttcaaaat-attgaaatttctttttc  \|\|\|\|\|\|\|\|\| \|\|\| \|\|\|\|\|\|\|\|\|\|\|\|\| cttcaaaattatt-aaatttctttttc  atttagatataaaaaaatag  \|\|\|\|\|\|\|\|\|\|\|\|\|\|\|\|\|\|\|\| atttagatataaaaaaatag |
| 2AX | 38191 | *Pseudomonas* phage Ab31 | *Pseudomonas* sp | cagaactgctcaaacttctccggcttcatgcg  \|\|\|\|\|\|\|\| \|\| \|\|\|\|\|\|\|\|\|\|\|\|\|\|\|\|\|\|\|\| cagaactgttcgaacttctccggcttcatgcg |
| 2AX | 34785 | *Pseudomonas* phage phiPSA1 | *Pseudomonas chlororaphis*  *Clostridium stercorarium*  *Pseudomonas chlororaphis* | tcccgctggttgccgatgggcgcgggg-gag  \|\|\|\|\|\|\|\|\|\|\|\|\|\|\|\|\|\|\|\|\|\|\|\|\|\|\| \|\|\| tcccgctggttgccgatgggcgcggggtgag  gccctggccgccgccga-a-aagcaga  \|\|\|\|\|\|\|\|\|\|\|\|\|\|\|\|\| \| \|\|\|\|\|\|\| gccctggccgccgccgacagaagcaga  aggctgaggtggttgctgatcatgctgcc  \|\|\|\|\|\|\|\|\|\|\| \|\|\|\|\|\|\|\|\|\|\|\| \|\|\|\| aggctgaggtgattgctgatcatggtgcc |
| 2AX | 6598 | *Burkholderia* phage BcepB1A | *Clostridium difficile* | taatatt-cttttatttgctagttcttcaa  \|\|\|\|\|\|\| \|\|\|\|\|\|\|\|\| \| \|\|\|\|\|\|\|\|\|\| taatatttcttttattt-caagttcttcaa |
| 2AX | 5137 | *Vibrio* phage vB_VpaM_MAR | *Calothrix* sp  *Desulfobacula toluolica*  *Clostridium difficile* | aaatgtatctttaata-ccagcacgactaataa  \|\|\| \|\|\|\|\|\|\|\|\|\|\|\| \|\|\|\|\|\|\| \|\| \|\|\|\|\| aaa-gtatctttaatacccagcac-ac-aataa  tagaaatgattaaatatctttaat  \|\|\|\|\|\|\|\|\|\|\|\|\|\|\|\|\|\| \|\|\|\|\| tagaaatgattaaatatc-ttaat  ttgaaaatagttacttttacgtaaaattt  \|\|\|\|\|\|\|\|\|\|\| \|\|\|\|\|\|\|\|\| \|\|\|\|\|\|\| ttgaaaatagtgacttttacgaaaaattt |
| 2F | 41737 | *Enterobacteria* phage HK446 | *Dokdonella koreensis* | accgccaccagcgccgattgcagttagc  \|\|\|\|\|\|\|\|\|\|\|\|\|\|\| \|\|\|\|\| \|\|\|\|\|\| accgccaccagcgcc-attgccgttagc |
| 2F | 32618 | *Ralstonia* phage RSY1 | *Caldicellulosiruptor lactoaceticus* | caaaaaatggggaaaagaccgaaa  \|\|\|\|\|\|\|\|\|\|\| \|\|\|\|\|\|\|\|\|\|\|\| caaaaaatggg-aaaagaccgaaa |
| 2F | 6653 | *Ralstonia* phage RSY1 | *Sulfolobus islandicus* | aagat-cagaagatttaaaatta  \|\|\|\|\| \|\|\|\|\|\|\|\|\|\|\|\|\|\|\|\|\| aagatgcagaagatttaaaatta |
| 2F | 5728 | *Rhodothermus* phage RM378 | *Sulfolobus islandicus* | gaaatagaacaaagctttaagcaattaa  \|\|\|\|\|\|\|\|\| \|\|\|\|\|\|\|\|\|\|\| \|\|\|\|\|\| gaaatagaaaaaagctttaag-aattaa |
